# Supplementary material for: Analytical and diagnostic performance characteristics of reverse-transcriptase loop-mediated isothermal amplification assays for dengue virus serotypes 1–4: A scoping review to inform potential use in portable molecular diagnostic devices
Source: PLOS Glob Public Health. 2023 Aug 8;3(8):e0002169. doi: 10.1371/journal.pgph.0002169 (PMC10409275; doi:10.1371/journal.pgph.0002169)
Supplement: Attachment — Submitted filename: PGPH-D-22-02107_R1_reviewer_LAMP Denv1to4.pdf [file pgph.0002169.s004.pdf]

PLOS Global Public Health

Analytical and diagnostic performance characteristics of reverse-transcriptase loop-mediated isothermal amplification assays for dengue virus serotypes 1-4: a scoping review to inform potential use in portable molecular diagnostic devices

--Manuscript Draft--

**Manuscript Number:**	PGPH-D-22-02107R1
**Article Type:**	Research Article
**Full Title:**	Analytical and diagnostic performance characteristics of reverse-transcriptase loop-mediated isothermal amplification assays for dengue virus serotypes 1-4: a scoping review to inform potential use in portable molecular diagnostic devices
**Short Title:**	Reviewing RT-LAMP assays for dengue virus serotypes 1-4
**Corresponding Author:**	Paul Arkell<br>Imperial College London<br>UNITED KINGDOM
**Order of Authors:**	Paul Arkell
	Dumrong Mairiang
	Adisak Songjaeng
	Kenny Malpartida-Cardenas
	Kerri Hill-Cawthorne
	Panisadee Avirutnan
	Pantelis Georgiou
	Alison Holmes
	Jesus Rodriquez-Manzano
**Keywords:**	diagnosis; Dengue; detection; LAMP; loop-mediated isothermal amplification; serotypes; Review
**Abstract:**	Background<br><br>Dengue is a mosquito-borne disease caused by dengue virus (DENV) serotypes 1-4 which affects 100-400 million adults and children each year. Reverse-transcriptase (RT) quantitative polymerase chain reaction (qPCR) assays are the current gold-standard in diagnosis and serotyping of infections, but their use in low-middle income countries (LMICs) has been limited due to laboratory infrastructure requirements. Loop-mediated isothermal amplification (LAMP) assays do not require thermocycling equipment and therefore have the potential to be deployed outside laboratories and/or miniaturised. This scoping literature review aimed to describe the analytical and diagnostic performance characteristics of previously developed serotype-specific dengue RT-LAMP assays and evaluate potential for use in portable molecular diagnostic devices.<br><br>Methods<br><br>A literature search in Medline was conducted. Studies were included if they were listed before 4th May 2022 (no prior time limit set) and described the development of any serotype-specific DENV RT-LAMP assay (‘original assays’) or described the further evaluation, adaption or implementation of these assays. Technical features, analytical and diagnostic performance characteristics were collected for each assay.<br><br>Results<br><br>Eight studies describing original RT-LAMP assays for dengue serotyping were identified. These were heterogenous in design and reporting. Assays’ lower limit of detection (LLOD) and linear range of quantification were comparable to RT-qPCR (with

Powered by Editorial Manager® and ProduXion Manager® from Aries Systems Corporation

	lowest reported values 2.2x101 and 1.98x102 copies/ml, respectively, for studies which quantified target RNA copies) and analytical specificity was high. When evaluated, diagnostic performance was also high, though reference diagnostic criteria varied widely, prohibiting comparison between assays. Fourteen studies using previously described assays were identified, including those where reagents were lyophilised or 'printed' into microfluidic channels and where several novel detection methods were used.<br><br>Discussion<br><br>Serotype-specific DENV RT-LAMP assays are high-performing and have potential to be used in portable molecular diagnostic devices if they can be integrated with sample extraction and detection methods. Standardised reporting of assay validation studies would be beneficial.
**Additional Information:**	
**Question**	**Response**
**Financial Disclosure**<br><br>Enter a financial disclosure statement that describes the sources of funding for the work included in this submission and the role the funder(s) played. This includes grants and any commercial funding of the work or authors.<br><br>This statement will be typeset if the manuscript is accepted for publication.<br><br>*Please review the submission guidelines and the instructions link below for detailed requirements and guidance.*	PA is funded by the Wellcome Trust (ref: 215688/Z/19/Z). KMP is funded by NIHR (ref: NIHR134694). KHC is funded by the Wellcome Trust (ref: 226691/Z/22/Z). PG and JRM are funded by Imperial College London. AH is funded by Imperial College London and the University of Liverpool. DM, AS and PAvirutan are funded by Mahidol University. The funders had no role in study design, data collection and analysis, decision to publish, or preparation of the manuscript.
**Competing Interests**<br><br>On behalf of all authors, disclose any competing interests that could be perceived to bias this work.<br><br>This statement will be typeset if the manuscript is accepted for publication.<br><br>*Please review the instructions link below and PLOS Global Public Health's competing interests policy to determine what information must be disclosed at submission.*	All authors declare there are no competing interests

**Data Availability**<br><br>Before publication, Authors are required to make fully available and without restriction all data underlying their findings. Please see our PLOS Data Policy page for detailed information on this policy.<br><br>A **Data Availability Statement**, detailing where the data can be accessed, is required at first submission. Insert your Data Availability Statement in the box below.<br><br>Please see the data reporting section of our submission guidelines for instructions on what you need to include in your Data Availability Statement.<br><br>This statement will be typeset if the manuscript is accepted for publication.<br><br>*PLOS allows rare exemptions to address legal and ethical concerns. If you have legal or ethical restrictions, please use the box below to detail these in full sentences for the Journal team to consider.*	Review article. All data is included in supporting information files

Manuscript
Click here to access/download;Manuscript;Manuscript_v8_clean.docx

Analytical and diagnostic performance characteristics of reverse-transcriptase loop-mediated isothermal amplification assays for dengue virus serotypes 1-4: a scoping review to inform potential use in portable molecular diagnostic devices

Paul Arkell[1,*], Dumrong Mairiang[2,3], Adisak Songjaeng[2,4], Kenny Malpartida-Cardenas[1], Kerri Hill-Cawthorne[1], Panisadee Avirutnan[2,3,4], Pantelis Georgiou[1,5], Alison Holmes[1,6], Jesus Rodriguez-Manzano[1]

1. *Centre for Antimicrobial Optimisation, Department of Infectious Disease, Imperial College London, Hammersmith Hospital, Du Cane Road, London, W12 0NN. United Kingdom.*
2. *Siriraj Center of Research Excellence in Dengue and Emerging Pathogens, Faculty of Medicine Siriraj Hospital, Mahidol University, Bangkok-noi, Bangkok 10700, Thailand.*
3. *Molecular Biology of Dengue and Flaviviruses Research Team, Medical Molecular Biotechnology Research Group, National Center for Genetic Engineering and Biotechnology (BIOTEC), National Science and Technology Development Agency (NSTDA), Bangkok 12120, Thailand.*
4. *Division of Dengue Hemorrhagic Fever Research, Department of Research and Development, Adulyadet Vikrom Building, 12th floor, Faculty of Medicine Siriraj Hospital, Mahidol University, Bangkok-noi, Bangkok 10700, Thailand.*
5. *Department of Electrical and Electronic Engineering, Imperial College London, Exhibition Road, SW7 2AZ. United Kingdom.*
6. *David Price Evans Global Health and Infectious Disease Research Group, University of Liverpool*

*** Corresponding Author:**
Dr Paul Arkell, Centre for Antimicrobial Optimisation, Imperial College London, Hammersmith Hospital, Du Cane Road, London.W12 0NN. United Kingdom.
Email: p.arkell@imperial.ac.uk

Keywords: Diagnosis, dengue, molecular detection, LAMP, loop-mediated isothermal amplification, serotypes, review

ABSTRACT

Background: Dengue is a mosquito-borne disease caused by dengue virus (DENV) serotypes 1-4 which affects 100-400 million adults and children each year. Reverse-transcriptase (RT) quantitative polymerase chain reaction (qPCR) assays are the current gold-standard in diagnosis and serotyping of infections, but their use in low-middle income countries (LMICs) has been limited due to laboratory infrastructure requirements. Loop-mediated isothermal amplification (LAMP) assays do not require thermocycling equipment and therefore have the potential to be deployed outside laboratories and/or miniaturised. This scoping literature review aimed to describe the analytical and diagnostic performance characteristics of previously developed serotype-specific dengue RT-LAMP assays and evaluate potential for use in portable molecular diagnostic devices.

Methods: A literature search in Medline was conducted. Studies were included if they were listed before 4th May 2022 (no prior time limit set) and described the development of any serotype-specific DENV RT-LAMP assay ('original assays') or described the further evaluation, adaption or implementation of these assays. Technical features, analytical and diagnostic performance characteristics were collected for each assay.

Results: Eight studies describing original RT-LAMP assays for dengue serotyping were identified. These were heterogenous in design and reporting. Assays' lower limit of detection (LLOD) and linear range of quantification were comparable to RT-qPCR (with lowest reported values $2.2x10^1$ and $1.98x10^2$ copies/ml, respectively, for studies which quantified target RNA copies) and analytical specificity was high. When evaluated, diagnostic performance was also high, though reference diagnostic criteria varied widely, prohibiting comparison between assays. Fourteen studies using previously described assays were identified, including those where reagents were lyophilised or 'printed' into microfluidic channels and where several novel detection methods were used.

Discussion: Serotype-specific DENV RT-LAMP assays are high-performing and have potential to be used in portable molecular diagnostic devices if they can be integrated with sample extraction and detection methods. Standardised reporting of assay validation studies would be beneficial.

BACKGROUND:

Dengue is a mosquito-borne disease caused by dengue virus serotypes 1-4 (DENV 1-4). International travel, urbanisation and climate change have contributed to increasing global incidence with up to half the world's population across 125 tropical and sub-tropical countries now at-risk.(1–3) There are an estimated 100-400 million infections annually which cause a spectrum of disease ranging from asymptomatic or mild, self-limiting symptoms to severe forms of the disease, including dengue haemorrhagic fever and dengue shock syndrome.(4,5) ‘Secondary infection’, when an individual is infected for a second (or subsequent) time in their life by a different serotype to their ‘primary infection’ is most likely to result in severe disease.(6–8) Therefore, shifts in the predominant circulating DENV serotype can be associated with outbreaks.(9) Better access to serotype-specific diagnostic testing for dengue may improve case-management, surveillance and disease control.(10)

The dengue diagnostic gap

Reverse-transcriptase polymerase chain reaction (RT-PCR) assays are the current gold-standard in diagnosis and serotyping of dengue infections.(11) These detect DENV ribonucleic acid (RNA) which is present in clinical samples from the onset of symptoms in both primary and secondary infection. A RT enzyme is used to synthesise complementary deoxyribonucleic acid (cDNA) from a target RNA sequence, and a DNA polymerase enzyme is used to amplify the cDNA. PCR primers can be designed to either bind regions of the DENV genome which are conserved across serotypes (resulting in a ‘generic’ dengue assay), or regions which are specific to an individual serotype (resulting in a serotype-specific assay).(12) PCR assays can quantify DENV RNA if performed in real-time PCR (RT-qPCR), and can be multiplexed with assays for other pathogens. (13,14) However, in all PCR assays, amplification of nucleic acid occurs during repeated cycles of heating and cooling to achieve precise denaturation and annealing temperatures, requiring significant laboratory infrastructure and a reliable power supply. They also require skilled operators and systems for quality and safety. As such, deploying PCR facility in many low-middle income countries (LMICs) is impractical. Patients in

most settings do not receive serotype-specific DENV testing and this may impact disease surveillance and control efforts. A multicentre observational study on the global availability of testing highlighted the diagnostic gap in LMICs, which particularly affects remote and regional areas.(15,16)

Loop-mediated isothermal amplification (LAMP) as a potential solution

Loop-mediated isothermal amplification (LAMP) is an isothermal nucleic acid amplification method first described in 2000 by Notomi et al.(17) When coupled with a reverse-transcriptase step (RT-LAMP), it can be used to detect RNA (Figure 2). Samples can be analysed directly, but usually undergo a nucleic acid extraction step to isolate and purify RNA, removing undesired components which may inhibit or otherwise affect the efficiency of the amplification reaction. LAMP uses a *Bacillus stearothermophilus* (*Bst*) DNA polymerase enzyme which possesses high autocycling strand displacement activity, allowing DNA synthesis to occur at a constant temperature (typically between 60-65 degrees Celsius). Multiple primers are used to create continuous loop structures during amplification. Primers may be designed to produce either generic or serotype-specific dengue assays. LAMP primers include forward and backward outer primers (F3 and B3, respectively), forward and backward inner primers (FIP and BIP), and forward and backward loop primers (FLP and BLP, which are not essential but can improve efficiency of the reaction). Amplified nucleic acids can be detected and/or quantified using various methods including visual inspection, turbidometry, gel electrophoresis, real-time monitoring using intercalating dyes and hybridisation with fluorescent probes, or non-fluorescence methods such electrochemical sensors.(18–21) Because LAMP assays do not require thermocycling equipment, they have long been considered potentially more suited to miniaturisation and/or deployment outside the laboratory setting than PCR assays, including for dengue and other neglected tropical diseases. (22,23)

Evaluating RT-LAMP assay performance

Studies which evaluate RT-LAMP assays may include measurement of various analytical performance characteristics (i.e. those which are inherent to the assay) and/or diagnostic (clinical) performance

characteristics (i.e. those which become relevant when the assay is used to detect a condition or disease).(24–26)

Analytical performance characteristics include lower limit of detection (LLOD, also known as 'analytical sensitivity' which is defined as the lowest concentration of a given substance that can be detected) and the linear range of quantification (also known as the 'reportable range', which for quantitative assays is the span of test result values over which the accuracy of the measurement can be verified). Additionally, analytical specificity (which is the ability of an assay to detect only the intended target and the absence of 'cross-reaction' with potentially interfering nucleic acids or specimen-related conditions) can be determined by interference studies which test 'no template controls' and/or samples containing potentially interfering substances or non-targeted biomarkers. If/when amplification does occur, various techniques can be used to verify authenticity of the DNA product, and hence analytical specificity of the assay. These include assessment of amplicon specificity using a specific restriction enzyme and agarose gel electrophoresis, amplification and melting curve studies, and nucleic acid sequencing techniques.(27,28) Assuring analytical specificity is particularly important in assay design because several phenomena including the formation of amplifiable primer-dimers and hairpin structures can lead to false-positivity.(29–31)

Diagnostic performance characteristics include diagnostic sensitivity (which is the ability of a test to correctly classify an individual as having a condition or disease, i.e. the number of true positive results as a fraction of the total number individuals with the condition or disease) and diagnostic specificity (which is the ability of a test to correctly classify an individual as not having a condition or disease, i.e. the number of true negative results as a fraction of the total number of individuals without the condition or disease). An important consideration for diagnostic accuracy studies is the choice of reference standard, which is used to compare the performance of the 'index test' against. This may be an alternative 'gold-standard' assay or may be based on validated clinical diagnostic criteria.(24)

Aim

The primary aim of this scoping review was to describe the technical features and performance characteristics of previously developed serotype-specific dengue RT-LAMP assays. The secondary aim was to evaluate their potential for use in portable molecular diagnostic devices.

METHODS

Methods for this scoping literature review were developed according to the Preferred Reporting Items for Systematic reviews and Meta-Analyses extension for Scoping Reviews guideline.(32) A literature search in Medline was conducted on 4th May 2022. The search strategy was constructed by two investigators from combinations of medical subject headings (MeSH) and keywords (Table 1). Results were imported into Covidence software and deduplicated.(33) Citations were reviewed at a title/abstract level for potential inclusion, then at full text level for inclusion by two authors. Discrepancies were resolved by discussion until consensus was reached. Citation lists from all studies examined at full text level, as well as those from all review articles identified by the original search, were also reviewed.

Studies were included if they were listed before 4th May 2022 (no prior time limit set) and fulfilled either of the following criteria: 1) Described one or more newly developed RT-LAMP assay which was designed to detect a single DENV serotype 1-4 ('original assays'); 2) Described the further evaluation, adaption or implementation of one these assays. Studies were excluded if they did not detail the primer sets which were used (either within the publication, supplementary material or by reference), if they described only generic dengue assays, or if they were not written in English language. All types of laboratory or clinical study design were eligible.

Data were collected from included studies according to a pre-determined proforma. This was designed based on the required performance characteristics which are needed before

implementation of laboratory-developed tests, as detailed in the Revised Clinical Laboratory Improvement Amendments (CLIA) of 2003(34) (analytical performance characteristics) and the updated Standards for Reporting Diagnostic Accuracy (STARD) statement of 2015 (diagnostic performance characteristics).(35) Where LLOD was given in plaque forming units (PFU) or RNA copies per microlitre, this was converted to PFU or RNA copies per reaction (by multiplying by the reported reaction volume). Technical features of the assay (extraction method, reaction mixture ingredients, primer sequences, incubation temperature and detection method) were also collected.

RESULTS

The database search retrieved 87 unique articles, of which 46 were excluded based on titles and abstracts. Two articles requiring full text review were also identified through review of citations. Forty-three full texts were therefore assessed for eligibility, of which 22 were included and 21 were excluded. The commonest reason for exclusion at full text review was 'assay was generic (i.e. not a serotype-specific assay)', which applied to 12 articles. Of the 22 studies included, 8 described original assays and 14 described the further evaluation, adaption, or implementation of a previously developed assay. A consort diagram is shown in Figure 1.

Technical features of original RT-LAMP assays

Eight sets of original RT-LAMP assays were developed by Parida et al (2005), Neeraja et al (2015), Hu et al (2015), Lau et al (2015), Yaren et al (2017), Kim et al (2018), Lopez-Jimena et al (2018) and Shoushtari et al (2021). (36–43) They were designed by obtaining sequences for dengue virus serotypes 1-4 from GenBank/NCBI database (7/8) or other sources (1/8). Various methods and software packages were used to identify potential template regions where sequences were conserved within sequences from the same serotype (but distinct from other serotypes and organisms) and assess possible secondary structures of primers. This included DNASIS software

(Hitachi, Japan, 2/8 studies), Primer-Explorer V5 (Eiken Chemical Co. LTD, Tokyo, Japan, 2/8), LAMP designer (Primer biosoft, America, 1/8), Primer-Explorer V3 (Eiken Chemical Co. LTD, Tokyo, Japan, 1/8), OligArch v2 (FfAME, Alachua, FL, 1/8), PrimerCompare v1 (FfAME, Alachua, FL, 1/8), and various R packages (1/8). Figure 2 shows a schematic of the dengue genome and the position of primer-binding for each serotype-specific RT-LAMP assay.

Out of the eight original assays, seven performed nucleic acid extraction using commercially available kits (most commonly QIAamp viral RNA mini kit, QIAGEN, Hilden Germany). All assays used commercially available preparations of *Bst* DNA polymerase and a reverse transcriptase enzyme (Avian Myeloblastosis Virus, AMV, or RTx reverse transcriptase). Some assays used commercially available LAMP reaction mixes, while others used bespoke mixes which included deoxynucleoside triphosphates, betaine, Tween 20, $(NH4)_2SO4$, $MgSO_4$ (or $MgCl_2$), KCl and Tris–HCl.

Analytical performance of original RT-LAMP assays

When determined in 7/8 studies, LLOD of assays was between $2.5x10^{-3}$ and $1.22x10^{0}$ plaque-forming unit (PFU)/reaction (for studies which quantified target in PFU) and between $2.2x10^{1}$ and $8.25x10^{2}$ copies/reaction (for studies which quantified target in RNA copies). However, some studies did not give detailed description of the method for quantification of viral particles and/or copies of template RNA used in LLOD experiments. Furthermore, it was sometimes not clear whether the cited concentrations referred to those of original samples, the elution buffer after nucleic acid extraction (i.e. the extract) or the final reaction mix. In this review, concentrations have been converted to 'per-reaction values', as best possible from the information available in manuscripts. When determined in 3/8 studies, the linear range of quantification went as low as $2.5x10^{0}$ PFU/reaction (for the study which quantified target in PFU) and as low as $1.98x10^{2}$ copies/reaction (for studies which quantified target in RNA copies), but similar difficulties interpreting quantification methods and cited concentrations were encountered.

Analytical specificity was usually assessed by testing viral particles of similar viruses (including discordant DENV serotypes and other flaviviruses), or their synthetic RNA or DNA templates. Virtually no incidents of non-specific amplification were reported across all studies. However, the total number of reactions conducted during these interference studies was often not reported, or was fewer than 10. Authenticity of the amplified product was evaluated using digestion with a specific restriction enzyme and agarose gel electrophoresis in 3/8 studies, nucleic acid sequencing in 3/8 studies, melting curve studies in 1/8 study, and was not done in 1/8 study. However, sometimes these data were not presented, and it was rarely clear whether authentication was undertaken for all experiments (i.e. every sample which was determined positive), or only a subset.

Diagnostic performance of original RT-LAMP assays

Assessment of diagnostic performance occurred in 6/8 studies. Most often panel of 'positive samples' were used, which had been tested in parallel using alternative methods (5/6 studies), or had been characterised previously and assigned 'dengue positive' based on clinical criteria (1/6 studies). A panel of 'negative samples' from healthy individuals were also used in most studies (5/6). RT-qPCR was most often used as a reference standard, but some studies did not clearly detail which test and/or clinical case definition was being used as a reference standard. In 1/6 study RT-LAMP testing resulted in significantly higher positivity than RT-qPCR, which was interpreted as superior diagnostic sensitivity but may also have been due to low diagnostic specificity.

Adaption of original RT-LAMP assays towards portable molecular diagnostic devices

Fourteen **studies** described the further evaluation, adaption, or implementation of a previously developed assay. None of these studies present a working portable molecular diagnostic device which has been deployed and thoroughly evaluated outside the laboratory setting. However, the following technological advances were presented:

Yamagishi et al (2017) adapted the Parida et al assay, analysing samples directly (i.e. without nucleic acid extraction) and loading amplified products into a portable MinION sequencer to determine the serotype. Sequencing was deemed necessary because erroneous LAMP signals were observed from negative control samples, and the workflow was ultimately used under 'field conditions' at a small clinic in Indonesia.(44)

Ganguli et al (2017) printed and dried Hu et al primers onto microfluidic channels in bespoke sample-processing and amplification chips. Amplification occurred on the chip while it is housed within a 3d-printed light-proof cradle and a smart phone was used to perform real time detection of fluorescence in each channel.(45)

Minero et al (2017) developed and applied two different detection methods using optomagnetic spectroscopy to the Lau et al assays. First, the interaction between biotinylated FIP or LF primers (which incorporate into amplicons during the LAMP reaction) and streptavidin-coated magnetic nanoparticles (included in the reaction mixture) was observed in real-time. Second, a method was devised to try and discriminate between 'true positive' and 'spurious' LAMP amplicons using a 3'-biotiylated 'loop-validating' DNA probe. In this study the authors highlight the common problem of spurious amplicons in LAMP assays and the importance of having a readout method which is not prone to detection of these. (46)

Priye et al (2017) described a 'quenching of unincorporated amplification signal reporters' (QUASR) technique which was used to multiplex the Lau et al DENV1 and DENV2 assays. The BIP primer was labelled with cyanine-5 and a short complimentary quenching probe was included, resulting in fluorescence upon cooling if specific amplification had occurred. The whole workflow was transferred into a 'smart phone-operated LAMP box' which included a heating module, an assay reaction housing module and an optical-detection/image-analysis module and gave a qualitative result for each target.(47)

Hin et al (2021) used The Lopez-Jimena assays in an automated device performing sample lysis, nucleic acid extraction, and up to 12 parallel LAMP reactions which are detected in real-time using fluorescence (the 'FeverDisk'). Analytical performance characteristics for the DENV assays in this format were not determined but specimens from some participants were tested positive.(48)

Kumar et al (2022) coated Prida et al primers with either biotin or digoxigenin, precipitated the amplified product using polyethylene glycol, and induced clumping with streptavidin- or anti-digoxigenin-coated magnetic particles. This produced a 'magnetic' assay which could be interpreted visually and multiplexed with another assay.(49)

Table 2 and appendix 1 summarise the studies included in this review.

DISCUSSION

This review identified eight studies describing original serotype-specific dengue RT-LAMP assays. All assays underwent evaluation of analytical performance with some also undergoing evaluation of diagnostic performance. However, studies were heterogenous in their design and reporting, and some omitted key experimental details. This made objective assessment and comparison of assays difficult and would likely affect attempts to replicate assays and verify findings. It is acknowledged that reports of assay development in academic literature is often a preliminary step, occurring before more rigorous efforts are made to achieve validation and accreditation. As such, authors may not be expected to fulfil requirements such as those set by CLIA for implementation of laboratory-developed tests (which were used as a template for data collection in this study). Nevertheless, standardised assay evaluation and more detailed reporting of performance would be beneficial.

When detailed, the method of nucleic acid extraction, ingredients of the reaction mix (apart from primers) and enzymes were broadly similar across original assays. However, incubation temperature, incubation duration and method for detecting the amplified product varied considerably. LLODs and

linear ranges were described for some assays and these analytical performance characteristics were comparable to those which are achievable with many RT-qPCR assays.(50) Analytical specificity was also reportedly good, with virtually no incidents of non-specific amplification being reported. However, the numbers of experiments conducted using no-template controls was generally low, and subsequent studies which used the same primer sets cite non-specificity as a particular reason for modifying incubation settings, primers and/or the detection method. Some studies interpreted higher positivity by RT-LAMP (index test) when compared to RT-qPCR (reference test) as evidence of superior RT-LAMP sensitivity, when in fact this could have been due to lower RT-LAMP specificity. Non-specific reactions are a feature of some nucleic acid amplification assays, including LAMP.(29–31) Therefore, assays must be designed carefully and evaluated thoroughly when this amplification chemistry is used. Further in-silico and in-vitro evaluation and modification of primer-sets may be useful, to inform and ensure their optimal performance in portable molecular diagnostic devices. Any future diagnostic accuracy studies which evaluate RT-LAMP assays (index tests) should clearly state which gold-standard assay or clinical diagnostic criteria (or composite thereof) is being used as a comparator (reference test). RT-qPCR, which is generally considered the highest performing single test for dengue infection, was the most common comparator assay in included studies. However, if novel RT-LAMP based assays are developed which are truly portable and can be used at the 'point-of-care' (including sample preparation, amplification, and detection steps), then diagnostic performance of the system as a whole could also be compared to lateral flow assays, which can be used in similar settings.

Assays went on to be used in 14 subsequent studies. These included studies where samples were tested directly (i.e. without any nucleic acid extraction prior to amplification). Assays which do not need sample preparation would be of huge benefit when considering their translation into portable diagnostic devices. However, the performance of 'direct LAMP' and superiority of LAMP assays over PCR assays in this regard is contentious.(51) They also included studies where reagents were lyophilised or 'printed' into microfluidic channels and those which used novel detection methods

including the use of smart phone cameras, electrochemical sensing, and sequencing (MinION). The restriction of this study to include only serotype-specific assays for detecting DENV 1-4 is a limitation, and it is acknowledged that other relevant technological advances are likely to have been made and demonstrated in other applications of LAMP-based diagnostics. Additionally, there may be other data on dengue RT-LAMP assays which have not been published or included in the Medline database or may have otherwise been missed by this scoping review's search strategy.

Overall, findings from this study show that serotype-specific RT-LAMP assays for dengue are high-performing. When coupled with novel methods for sample preparation and detection, these assays may ultimately lead to portable molecular diagnostic devices which could be used across tropical and sub-tropical regions where dengue is endemic.

ACKNOWLEDGMENTS

This work was supported by the Department of Health and Social Care-funded Centre for Antimicrobial Optimisation (CAMO) at Imperial College London; and the Wellcome Trust Innovator Award (Grant ref: 215688/Z/19/Z). AH and JRM are affiliated with the NIHR Health Protection Research Unit (HPRU) in Healthcare Associated Infections and Antimicrobial Resistance at Imperial College London in partnership with the UK Health Security Agency, in collaboration with, Imperial Healthcare Partners, the University of Cambridge and the University of Warwick. The views expressed in this publication are those of the authors and not necessarily those of the NHS, the National Institute for Health Research, the Department of Health and Social Care, or the UK Health Security Agency. AH is a National Institute for Health Research (NIHR) Senior Investigator and she is Chair of the David Price Evans Global Health and Infectious Diseases Research Group at the University of Liverpool.

REFERENCES

1. Zeng Z, Zhan J, Chen L, Chen H, Cheng S. Global, regional, and national dengue burden from 1990 to 2017: A systematic analysis based on the global burden of disease study 2017. EClinicalMedicine [Internet]. 2021 Feb 1 [cited 2022 Jun 24];32. Available from: https://pubmed.ncbi.nlm.nih.gov/33681736/

2. Brady OJ, Gething PW, Bhatt S, Messina JP, Brownstein JS, Hoen AG, et al. Refining the global spatial limits of dengue virus transmission by evidence-based consensus. PLoS Negl Trop Dis [Internet]. 2012 [cited 2022 Jun 24];6(8). Available from: https://pubmed.ncbi.nlm.nih.gov/22880140/

3. Messina JP, Brady OJ, Golding N, Kraemer MUG, Wint GRW, Ray SE, et al. The current and future global distribution and population at risk of dengue. Nat Microbiol. 2019 Sep 1;4(9):1508–15.

4. Guzman MG, Gubler DJ, Izquierdo A, Martinez E, Halstead SB. Dengue infection. Nat Rev Dis Primer. 2016 Aug 18;2(1):1–25.

5. Bhatt S, Gething PW, Brady OJ, Messina JP, Farlow AW, Moyes CL, et al. The global distribution and burden of dengue. Nature. 2013 Apr 25;496(7446):504–7.

6. Montoya M, Gresh L, Mercado JC, Williams KL, Vargas MJ, Gutierrez G, et al. Symptomatic Versus Inapparent Outcome in Repeat Dengue Virus Infections Is Influenced by the Time Interval between Infections and Study Year. PLoS Negl Trop Dis [Internet]. 2013 [cited 2022 Nov 9];7(8). Available from: https://pubmed.ncbi.nlm.nih.gov/23951377/

7. De Alwis R, Williams KL, Schmid MA, Lai CY, Patel B, Smith SA, et al. Dengue Viruses Are Enhanced by Distinct Populations of Serotype Cross-Reactive Antibodies in Human Immune Sera. PLoS Pathog. 2014 Oct 1;10(10):1004386.

8. de Alwis R, Beltramello M, Messer WB, Sukupolvi-Petty S, Wahala WMPB, Kraus A, et al. In-depth analysis of the antibody response of individuals exposed to primary dengue virus infection. PLoS Negl Trop Dis [Internet]. 2011 Jun [cited 2022 Dec 12];5(6). Available from: https://pubmed.ncbi.nlm.nih.gov/21713020/

9. Lee KS, Lai YL, Lo S, Barkham T, Aw P, Ooi PL, et al. Dengue virus surveillance for early warning, Singapore. Emerg Infect Dis. 2010 May;16(5):847–9.

10. Rodriguez-Manzano J, Chia PY, Yeo TW, Holmes A, Georgiou P, Yacoub S. Improving Dengue Diagnostics and Management Through Innovative Technology. Curr Infect Dis Rep. 2018 Aug 1;20(8):1–8.

11. Muller DA, Depelsenaire ACI, Young PR. Clinical and laboratory diagnosis of dengue virus infection. J Infect Dis. 2017;215(Suppl 2):S89–95.

12. Alm E, Lindegren G, Falk KI, Lagerqvist N. One-step real-time RT-PCR assays for serotyping dengue virus in clinical samples. BMC Infect Dis [Internet]. 2015 Nov 2 [cited 2022 Nov 9];15(1). Available from: /pmc/articles/PMC4630907/

13. Morsy S, Hashan MR, Hieu TH, Mohammed AT, Elawady SS, Ghosh P, et al. The association between dengue viremia kinetics and dengue severity: A systemic review and meta-analysis. Rev Med Virol. 2020 Nov 1;30(6):1–10.

14. Manabe YC, Betz J, Jackson O, Asoala V, Bazan I, Blair PW, et al. Clinical evaluation of the BioFire Global Fever Panel for the identification of malaria, leptospirosis, chikungunya, and dengue from

whole blood: a prospective, multicentre, cross-sectional diagnostic accuracy study. Lancet Infect Dis. 2022 Sep 1;22(9):1356–64.

15. Fleming KA, Horton S, Wilson ML, Atun R, DeStigter K, Flanigan J, et al. The Lancet Commission on diagnostics: transforming access to diagnostics. The Lancet. 2021 Nov 27;398(10315):1997–2050.

16. Yadav H, Shah D, Sayed S, Horton S, Schroeder LF. Availability of essential diagnostics in ten low-income and middle-income countries: results from national health facility surveys. Lancet Glob Health. 2021 Nov 1;9(11):e1553–60.

17. Notomi T, Okayama H, Masubuchi H, Yonekawa T, Watanabe K, Amino N, et al. Loop-mediated isothermal amplification of DNA. Nucleic Acids Res. 2000;28(12):e63.

18. Zhang X, Lowe SB, Gooding JJ. Brief review of monitoring methods for loop-mediated isothermal amplification (LAMP). Biosens Bioelectron. 2014 Nov 15;61:491–9.

19. Moehling TJ, Choi G, Dugan LC, Salit M, Meagher RJ. LAMP Diagnostics at the Point-of-Care: Emerging Trends and Perspectives for the Developer Community. https://doi.org/101080/1473715920211873769. 2021;21(1):43–61.

20. Moser N, Yu LS, Rodriguez Manzano J, Malpartida-Cardenas K, Au A, Arkell P, et al. Quantitative detection of dengue serotypes using a smartphone-connected handheld lab-on-chip platform. Front Bioeng Biotechnol. 2022 Sep 15;10:892853–892853.

21. Rodriguez-Manzano J, Karymov MA, Begolo S, Selck DA, Zhukov DV, Jue E, et al. Reading Out Single-Molecule Digital RNA and DNA Isothermal Amplification in Nanoliter Volumes with Unmodified Camera Phones. ACS Nano. 2016 Mar 22;10(3):3102–13.

22. Njiru ZK. Loop-mediated isothermal amplification technology: Towards point of care diagnostics. PLoS Negl Trop Dis [Internet]. 2012 Jun [cited 2022 Nov 9];6(6). Available from: https://pubmed.ncbi.nlm.nih.gov/22745836/

23. Mori A, Pomari E, Deiana M, Perandin F, Caldrer S, Formenti F, et al. Molecular techniques for the genomic viral RNA detection of West Nile, Dengue, Zika and Chikungunya arboviruses: a narrative review. Expert Rev Mol Diagn. 2021;21(6):591–612.

24. Burd EM. Validation of laboratory-developed molecular assays for infectious diseases. Clin Microbiol Rev. 2010;23(3):550–76.

25. Peeling RW, Artsob H, Pelegrino JL, Buchy P, Cardosa MJ, Devi S, et al. Evaluation of diagnostic tests: Dengue. Nat Rev Microbiol. 2010;8(12):S30–8.

26. Saah AJ, Hoover DR. “Sensitivity” and “specificity” reconsidered: The meaning of these terms in analytical and diagnostic settings. Ann Intern Med. 1997;126(1):91–4.

27. Kreitmann L, Miglietta L, Xu K, Malpartida-Cardenas K, D’Souza G, Kaforou M, et al. Next-generation molecular diagnostics: Leveraging digital technologies to enhance multiplexing in real-time PCR. TrAC Trends Anal Chem. 2023 Mar 1;160:116963.

28. Malpartida-Cardenas K, Miglietta L, Peng T, Moniri A, Holmes A, Georgiou P, et al. Single-channel digital LAMP multiplexing using amplification curve analysis. Sens Diagn. 2022;1(3):465–8.

29. Meagher RJ, Priye A, Light YK, Huang C, Wang E. Impact of primer dimers and self-amplifying hairpins on reverse transcription loop-mediated isothermal amplification detection of viral RNA. Analyst. 2018 Apr 21;143(8):1924–33.

30. Jang MJ, Kim S. Inhibition of Non-specific Amplification in Loop-Mediated Isothermal Amplification via Tetramethylammonium Chloride. Biochip J. 2022 Sep 1;16(3):326–33.

31. Özay B, McCalla SE. A review of reaction enhancement strategies for isothermal nucleic acid amplification reactions. Sens Actuators Rep. 2021 Nov 1;3:100033.

32. Tricco AC, Lillie E, Zarin W, O'Brien KK, Colquhoun H, Levac D, et al. PRISMA extension for scoping reviews (PRISMA-ScR): Checklist and explanation. Ann Intern Med. 2018;169(7):467–73.

33. Covidence - Better systematic review management [Internet]. [cited 2021 Aug 25]. Available from: https://www.covidence.org/

34. Department of Health and Human Services Centers for Medicare & Medicaid Services Centers for Disease Control and Prevention 42 CFR Part 493 Medicare, Medicaid, and CLIA Programs; Laboratory Requirements Relating to Quality Systems and Certain Personnel Qu. 2003 [cited 2022 Dec 12]; Available from: www.access.gpo/nara/index.html.

35. Bossuyt PM, Reitsma JB, Bruns DE, Gatsonis CA, Glasziou PP, Irwig L, et al. STARD 2015: An updated list of essential items for reporting diagnostic accuracy studies. The BMJ [Internet]. 2015 Oct 28 [cited 2022 Oct 21];351. Available from: /pmc/articles/PMC4623764/

36. Parida M, Horioke K, Ishida H, Dash PK, Saxena P, Jana AM, et al. Rapid detection and differentiation of dengue virus serotypes by a real-time reverse transcription-loop-mediated isothermal amplification assay. J Clin Microbiol. 2005 Jun;43(6):2895–903.

37. Neeraja M, Lakshmi V, Lavanya V, Priyanka EN, Parida MM, Dash PK, et al. Rapid detection and differentiation of dengue virus serotypes by NS1 specific reverse transcription loop-mediated isothermal amplification (RT-LAMP) assay in patients presenting to a tertiary care hospital in Hyderabad, India. J Virol Methods. 2015 Jan 1;211:22–31.

38. Hu SF, Li M, Zhong LL, Lu SM, Liu ZX, Pu JY, et al. Development of reverse-transcription loop-mediated isothermal amplification assay for rapid detection and differentiation of dengue virus serotypes 1-4. BMC Microbiol [Internet]. 2015 [cited 2022 Dec 12];15(1). Available from: https://pubmed.ncbi.nlm.nih.gov/26572227/

39. Lau YL, Lai MY, Teoh BT, Abd-Jamil J, Johari J, Sam SS, et al. Colorimetric detection of dengue by single tube reverse-transcription-loop-mediated isothermal amplification. PLoS ONE [Internet]. 2015 Sep 18 [cited 2022 Feb 11];10(9). Available from: https://pubmed.ncbi.nlm.nih.gov/26384248/

40. Yaren O, Alto BW, Gangodkar PV, Ranade SR, Patil KN, Bradley KM, et al. Point of sampling detection of Zika virus within a multiplexed kit capable of detecting dengue and chikungunya. BMC Infect Dis [Internet]. 2017 Apr 20 [cited 2022 Dec 12];17(1). Available from: https://pubmed.ncbi.nlm.nih.gov/28427352/

41. Kim JG, Baek SH, Kim S, Kim HI, Lee SW, Phan LMT, et al. Rapid discriminative detection of dengue viruses via loop mediated isothermal amplification. Talanta. 2018 Dec 1;190:391–6.

42. Lopez-Jimena B, Bekaert M, Bakheit M, Frischmann S, Patel P, Simon-Loriere E, et al. Development and validation of four one-step real-time RT-LAMP assays for specific detection of each dengue virus serotype. PLoS Negl Trop Dis. 2018;12(5):1–22.

43. Shoushtari M, Salehi-Vaziri M, Roohvand F, Arashkia A, Jalali T, Azadmanesh K. Taguchi array optimization of the reverse transcription loop-mediated isothermal amplification (RT-LAMP) assay for sensitive and rapid detection of dengue virus serotype 2. Biotechnol Lett. 2021 Nov 1;43(11):2149–60.

44. Yamagishi J, Runtuwene LR, Hayashida K, Mongan AE, Thi LAN, Thuy LN, et al. Serotyping dengue virus with isothermal amplification and a portable sequencer. Sci Rep [Internet]. 2017 Dec 1 [cited 2022 Dec 12];7(1). Available from: https://pubmed.ncbi.nlm.nih.gov/28615658/

45. Ganguli A, Ornob A, Yu H, Damhorst GL, Chen W, Sun F, et al. Hands-free smartphone-based diagnostics for simultaneous detection of Zika, Chikungunya, and Dengue at point-of-care. Biomed Microdevices [Internet]. 2017 Dec 1 [cited 2022 Dec 12];19(4). Available from: https://pubmed.ncbi.nlm.nih.gov/28831630/

46. Minero GAS, Nogueira C, Rizzi G, Tian B, Fock J, Donolato M, et al. Sequence-specific validation of LAMP amplicons in real-time optomagnetic detection of Dengue serotype 2 synthetic DNA. Analyst. 2017 Sep 21;142(18):3441–50.

47. Priye A, Bird SW, Light YK, Ball CS, Negrete OA, Meagher RJ. A smartphone-based diagnostic platform for rapid detection of Zika, chikungunya, and dengue viruses. Sci Rep [Internet]. 2017 Mar 20 [cited 2022 Feb 11];7. Available from: /pmc/articles/PMC5357913/

48. Hin S, Lopez-Jimena B, Bakheit M, Klein V, Stack S, Fall C, et al. Fully automated point-of-care differential diagnosis of acute febrile illness. PLoS Negl Trop Dis. 2021 Feb 1;15(2):1–24.

49. Kumar S, Sharma S, Kumari S, Pande V, Savargaonkar D, Anvikar AR. Magnetic Multiplex Loop Mediated Isothermal Amplification (MM-LAMP) technique for simultaneous detection of dengue and chikungunya virus. J Virol Methods [Internet]. 2022 Feb 1 [cited 2022 Dec 12];300. Available from: https://pubmed.ncbi.nlm.nih.gov/34896457/

50. Songjaeng A, Thiemmeca S, Mairiang D, Punyadee N, Kongmanas K, Hansuealueang P, et al. Development of a Singleplex Real-Time Reverse Transcriptase PCR Assay for Pan-Dengue Virus Detection and Quantification. Viruses [Internet]. 2022 Jun 1 [cited 2022 Dec 12];14(6). Available from: https://pubmed.ncbi.nlm.nih.gov/35746742/

51. Wilson-Davies ESW, Mahanama AIK, Samaraweera B, Ahmed N, Friar S, Pelosi E. Concerns regarding the sensitivity of the OptiGene direct SARS-CoV-2 LAMP assay and its suitability for use in at-risk groups and hospital staff. J Infect. 2021 Feb 1;82(2):282–327.

SUPPORTING INFORMATION CAPTIONS

S1 Table: PRISMA-ScR checklist

S1 Data: Data collected from individual papers describing original RT-LAMP assays

FUNDING

PA is funded by the Wellcome Trust (ref: 215688/Z/19/Z). KMP is funded by NIHR (ref: NIHR134694). KHC is funded by the Wellcome Trust (ref: 226691/Z/22/Z). PG and JRM are funded by Imperial College London. AH is funded by Imperial College London and the University of Liverpool. DM, AS and PAvirutan are funded by Mahidol University. The funders had no role in study design, data collection and analysis, decision to publish, or preparation of the manuscript.

COMPETING INTERESTS

All authors declare there are no competing interests

Table 1: Search terms

1	exp Dengue/ or exp Dengue Virus/
2	dengue.mp.
3	loop mediated.mp.
4	isothermal amplification.mp.
5	LAMP.mp.
6	1 or 2
7	3 or 4 or 5
8	6 and 7

Table 2: Summary of studies describing original RT-LAMP assays for dengue virus serotypes 1-4

**Publication (year)**	**Assay details**					**Analytical performance**			**Diagnostic performance**				**Subsequent publications (year)**
	**Extraction method**	**Reaction mixture**	**Incubation**	**Detection**	**Target (gene)**	**Lower limit-of-detection**	**Specificity: other organisms**	**Specificity: examination of amplified product**	**Specimens**	**Reference standard**	**Sensitivity**	**Specificity**	
Parida et al (2005)	QIAamp viral RNA mini kit (QIAGEN, Hilden Germany)	Loopamp DNA amplification kit (Eiken Chemical Co. Ltd., Japan)	63.0 degrees Celsius for 60 mins (but determined positive at 30 mins)	Visual inspection (+/- addition of SYBR Green I) and real-time monitoring of turbidity and agarose gel analysis*	DENV1 (3' UTR)<br>DENV2 (3' UTR)<br>DENV3 (3' UTR)<br>DENV4 (3' UTR)	1 PFU/ml (=2.5x10^-2 PFU/reaction)<br>0.1 PFU/ml (=2.5x10^-3 PFU/reaction)<br>0.1 PFU/ml (=2.5x10^-3 PFU/reaction)<br>0.1 PFU/ml (=2.5x10^-3 PFU/reaction)	JEV, WNV or SLEV templates - no amplification	Restriction enzyme digestion + agarose gel electrophoresis - product sizes in good agreement with predicted. Further confirmation with sequencing - nucleotide sequences matched target	83 serum samples:<br>- 25 confirmed dengue cases<br>- 38 suspected dengue cases<br>- 20 healthy individuals	Considered positive if: Conventional RT-PCR (+) OR nested RT-PCR (+) OR virus isolation (+)	31/31 (100.0%)	20/20 (100.0%)	Li et al (2011)<br>Chagan-Yasutan et al (2013)<br>Lo et al (2013)<br>Yamagishi et al (2017)<br>Kumar et al (2022)<br>Gaber et al (2022)
Neeraja et al (2015)	QIAamp viral RNA mini kit (QIAGEN, Hilden Germany)	Isothermal Master Mix ISO-001 (Optigene, U.K.)	63.0 degrees Celsius for 35 minutes	Visual inspection (+/- addition of SYBR Green I) and agarose gel analysis*	DENV1 (NS1)<br>DENV2 (NS1)<br>DENV3 (NS1)<br>DENV4 (NS1)	N/A	Other flaviviruses (sic) including JEV, WNV, HCV and CHIKV - no amplification	Restriction enzyme digestion + agarose gel electrophoresis - product sizes in good agreement with predicted. Further confirmation using sequencing - results not reported in manuscript	300 serum or plasma samples:<br>- 250 dengue cases<br>- 50 healthy individuals	Considered positive if: RT-qPCR (+)	140/140 (100.0%)**	152/160 (95.0%)**	Dave et al (2022)
Hu et al (2015)	QIAamp viral RNA mini kit (QIAGEN, Hilden Germany)	Bespoke mix	63.0 degrees Celsius for 45 minutes	Visual inspection (+/- addition of SYBR Green I) and real-time monitoring of flourescence (SYBR Green I) and agarose gel analysis*	DENV1 (NS2A)<br>DENV2 (NS4B)<br>DENV3 (NS4A)<br>DENV4 (3'UTR)	1x10^1 copies/uL (=2.5x10^2 copies/reaction)<br>1x10^1 copies/uL (=2.5x10^2 copies/reaction)<br>1x10^1 copies/uL (=2.5x10^2 copies/reaction)<br>1x10^1 copies/uL (=2.5x10^2 copies/reaction)	JEV, YFV, HSV and Epstein-Barr virus x10 times - no amplification	Restriction enzyme digestion + agarose gel electrophoresis - images shown in manuscript. Further confirmation using sequencing - 'specificity of amplification confirmed'	210 serum samples:<br>- 190 confirmed dengue cases<br>- 20 healthy individuals	Considered positive if: 'confirmed to be infected by dengue by clinical diagnosis'	50/50 (100.0%)<br>59/60 (98.3%)<br>40/40 (100%)<br>39/40 (97.5%)	20/20 (100.0%)<br>20/20 (100.0%)<br>20/20 (100%)<br>20/20 (100%)	Ganguli et al ()

Lau et al (2015)	QIAamp viral RNA mini kit (QIAGEN, Hilden Germany)	Loopamp RNA amplification kit (Eiken Chemical Co. Ltd., Japan)	65.0 degrees Celsius for 30 min (DENV1-3 assays) or 45 min (DENV4 assay)	Visual inspection (+ HNB dye, Sigma, USA) and real-time monitoring of turbidity*	DENV1 (3' NCR)<br>DENV2 (3' NCR)<br>DENV3 (3' NCR)<br>DENV4 (3' NCR)	The detection limit of RT-LAMP for 3'-NCR was as low as ten copies (=2.5x10^2 copies/reaction)	JEV, CHIKV and Sindbis virus - no amplification	Agarose gel electrophoresis - typical DNA ladder observed (though analysis of amplicon size using restriction enzyme not reported)	213 serum samples<br>- 189 suspected dengue cases<br>- 24 healthy individuals	Considered positive if 2 or more of the following were true: RT-qPCR (+), ELISA (+), RT-LAMP (+).	115/115 (100%)	98/98 (100%)	Minero et al (2017)<br>Priye et al (2017)<br>Meagher et al (2018)<br>Sigera et al (2019)
Yaren et al (2017)	Unclear	Bespoke mix	65.0 degrees Celsius for 60-90 minutes	Real-time detection of fluorescence (TAMARA-labelled LB or LF probe) then modification to include 'target specific strand-displaceable probe' (fluorescence detected by cell phone camera).	DENV1 (NS5)	1.22 PFU per assay (=1.22x10^0 PFU/reaction)	ZIKV and CHIKV RNA - no amplification	Agarose gel electrophoresis - typical DNA ladder observed (though analysis of amplicon size using restriction enzyme not reported)	N/A	N/A	N/A	N/A	Yaren et al (2018)
Kim et al (2018)	QIAamp viral RNA mini kit (QIAGEN, Hilden Germany)	Bespoke mix	69.7 (DENV1 assay), 65.0 (DENV2 assay) or 66.5 (DENV4 assay) degrees Celsius for 40 minutes	Visual inspection (UV light illumination)	DENV1 (E)<br>DENV2 (NS1)<br>DENV4 (PrM)	33 copies / uL (=8.25x10^2 copies/reaction)<br>3.55 copies / uL (=8.88x10^1 copies/reaction)<br>9.06 copies / uL (2.27x10^2 copies/reaction)	DENV3, norovirus, rotavirus and bovine viral diarrhea - no amplification	Agarose gel electrophoresis - typical DNA ladder observed (though analysis of amplicon size using restriction enzyme not reported)	N/A	N/A	N/A	N/A	
Lopez-Jimena et al (2018)	Various commercially available extraction methods depending on source of samples/viruses	Bespoke mix	64.0 degrees Celsius for 45 min (DENV1 assay), 90 min (DENV2 assay), 75 min (DENV3 assay) or 50 min (DENV4 assay)	Real-time detection of fluorescence	DENV1 (various)<br>DENV2 (various)<br>DENV3 (various)	22 RNA molecules per reaction (=2.2x10^1 copies/reaction)<br>542 RNA molecules per reaction (=5.42x10^2 copies/reaction)<br>92 RNA molecules per reaction (9.2x10^1 copies/reaction)	ZIKV, YFV, WNV, Ntaya virus, *S. typhi*, *S. paratyphi*, *S. pneumoniae* and *P. falciparum* - no amplification	Melting curve analysis - single peak temperatures indicated specific amplification	78 samples:<br>- 42 imported blood/serum samples<br>- 36 imported RNA extracts	Considered positive if: RT-qPCR (+)	Initially sensitivity = 17/24 (70.8%), then false-negative samples re-extracted re-run and sensitivity = 23/24 (95.8%)**	7/7 (100%)**	Hin et al (2021)

					DENV4 (various)	197 RNA molecules per reaction (=1.97x10^2 copies/reaction)							
Shoushtari et al (2021)	QIAamp viral RNA mini kit (QIAGEN, Hilden Germany)	Bespoke mix	65.0 degrees Celsius for 60 minutes	Agarose gel analysis	DENV2 (C-PrM)	100 RNA copies per reaction (=1x10^2 copies/reaction)	DENV1, DENV3, DENV4, WNV, YFV, ZIKV RNA (and serum from hepatitis C patient) - no amplification	N/A	31 serum samples<br>- 20 dengue cases<br>- 11 healthy individuals	Considered positive if: RT-qPCR (+)	15/15 (100%)	Results for 11 healthy sera not presented	

Abbreviations: DENV = dengue virus, PFU = plaque forming units, JEV = Japanese encephalitis virus, WNV = West Nile virus, SLE = St Louis encephalitis virus, HCV = hepatitis C virus, CHIKV = chikungunya virus, HSV = herpes simplex virus, ZIKV = Zika virus, RT-PCR = reverse-transcriptase polymerase chain reaction, ELISA = enzyme-linked immunosorbent assay, RT-LAMP = reverse transcriptase loop-mediated isothermal amplification

* Studies frequently described more than one method for detecting amplified products of RT-LAMP. However, it was sometimes not clear how discrepant results were handled in analysis of assay analytical and diagnostic performance
** Multiple alternative analyses are reported in the manuscript

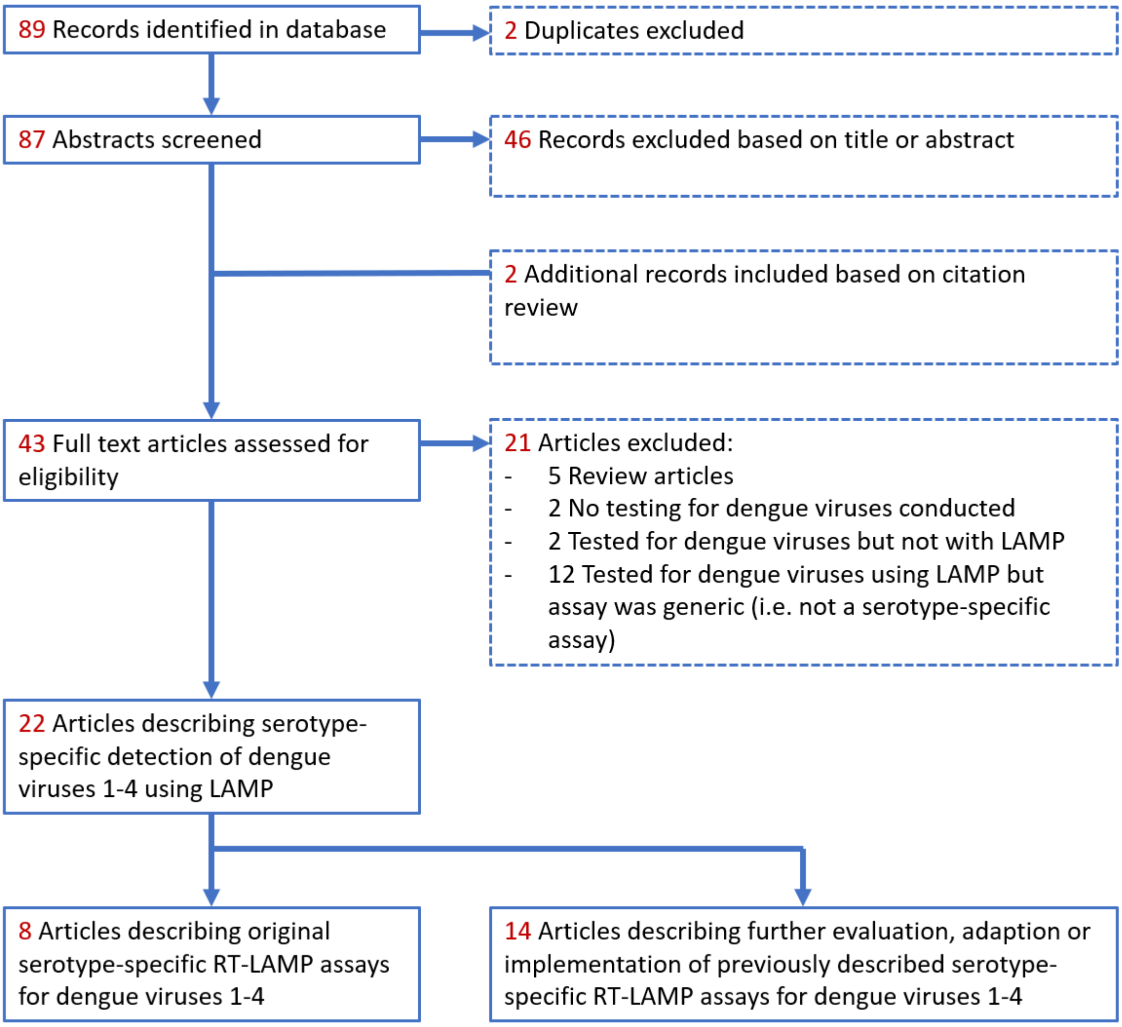

Figure 1: Workflow showing the assessment of articles and their inclusion in this review

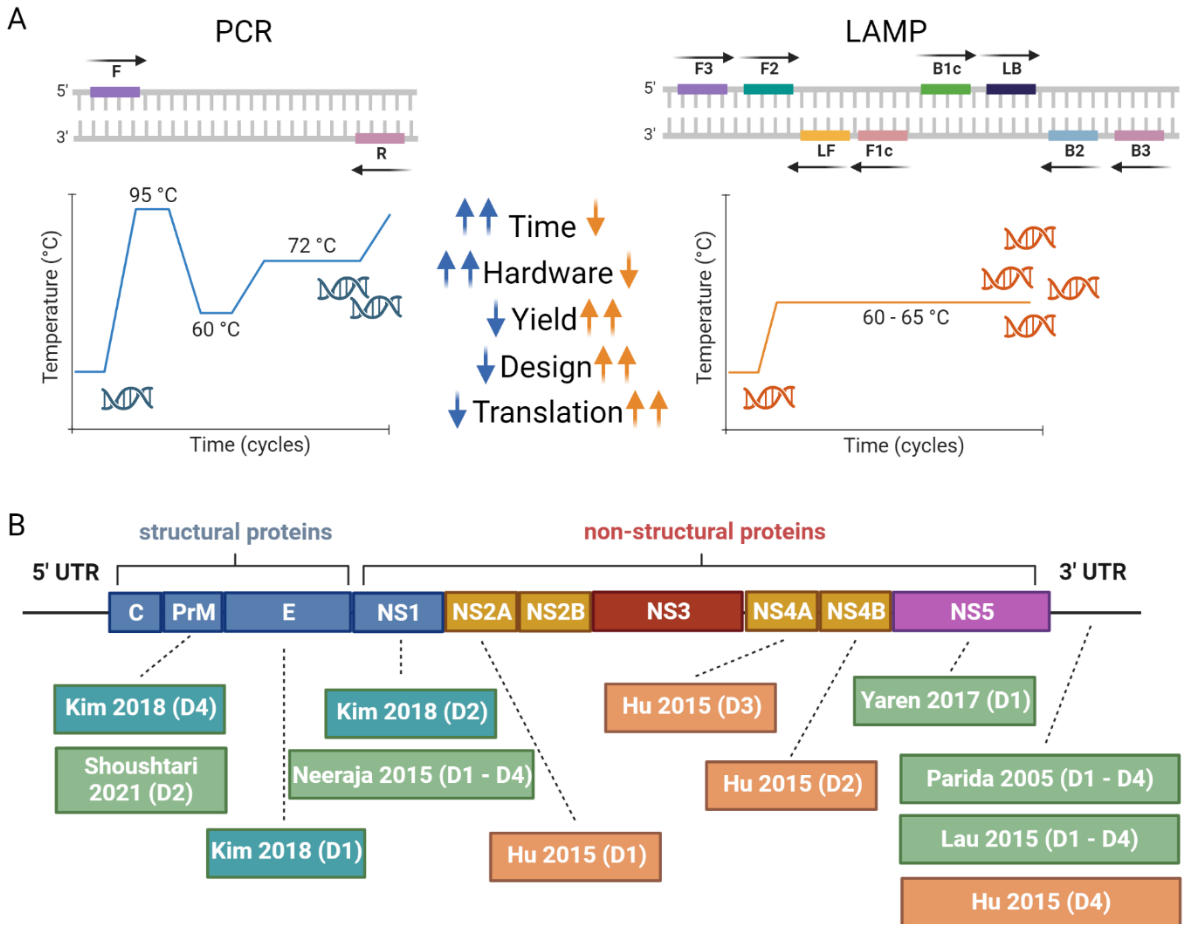

Figure 2: (A) Comparison between polymerase chain reaction (PCR) and loop-mediated isothermal amplification (LAMP). Primer binding regions are shown at the top, thermal cycling conditions at the bottom, and differences between the methods in the text where arrows in orange refers to LAMP and in blue to PCR. (B) Schematic of dengue genome showing the position of primer-binding in published serotype-specific RT-LAMP assays.

Figure 1: Workflow showing the assessment of articles and their inclusion in this review

Click here to access/download;Figure;Figure 1 v2.tif

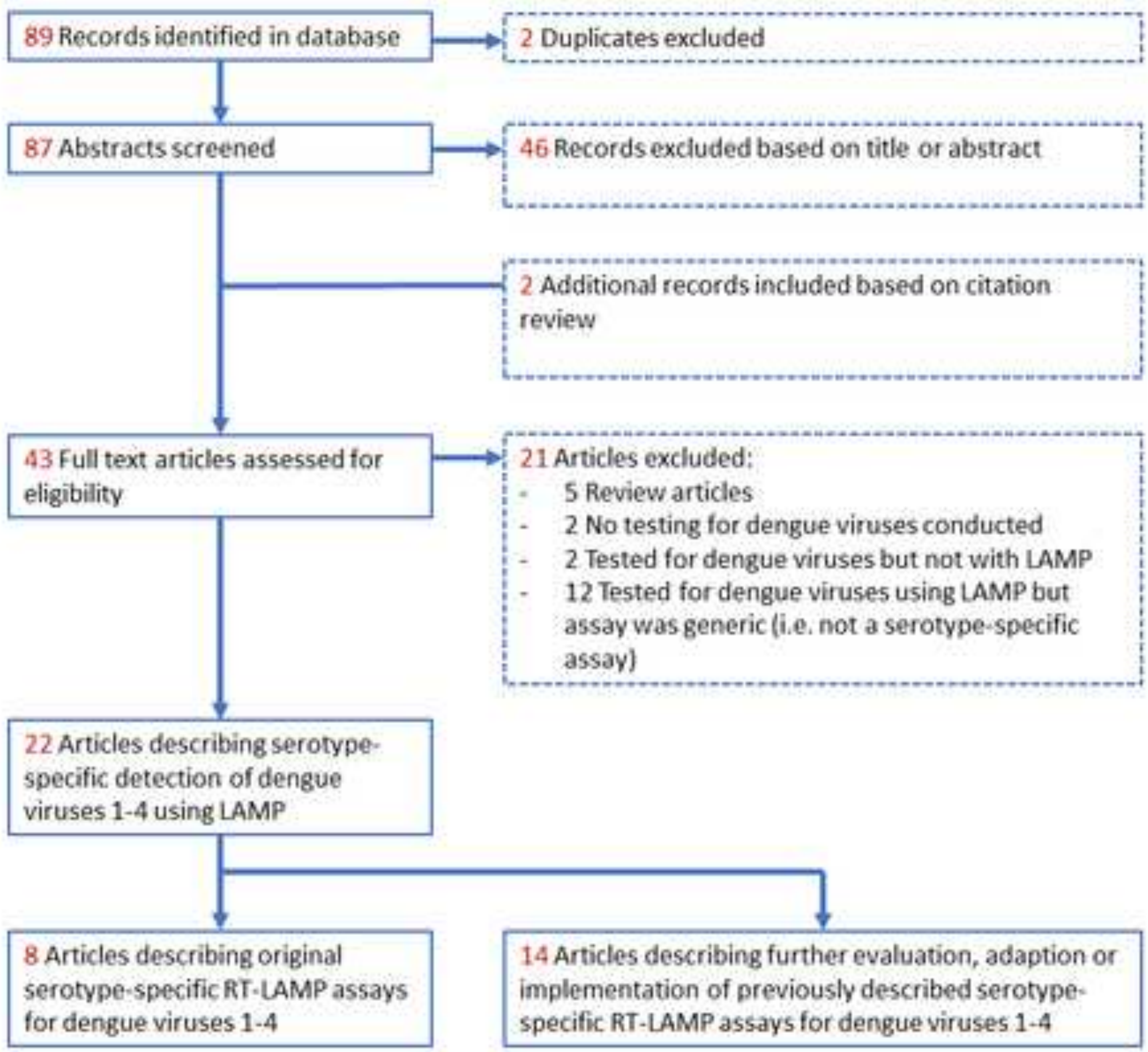

Figure 2: (A) Comparison between polymerase chain reaction (PCR) and loop-mediated isothermal amplification (LAMP). Primer binding regions are shown at the

Click here to access/download;Figure;Figure_2_v2.tiff

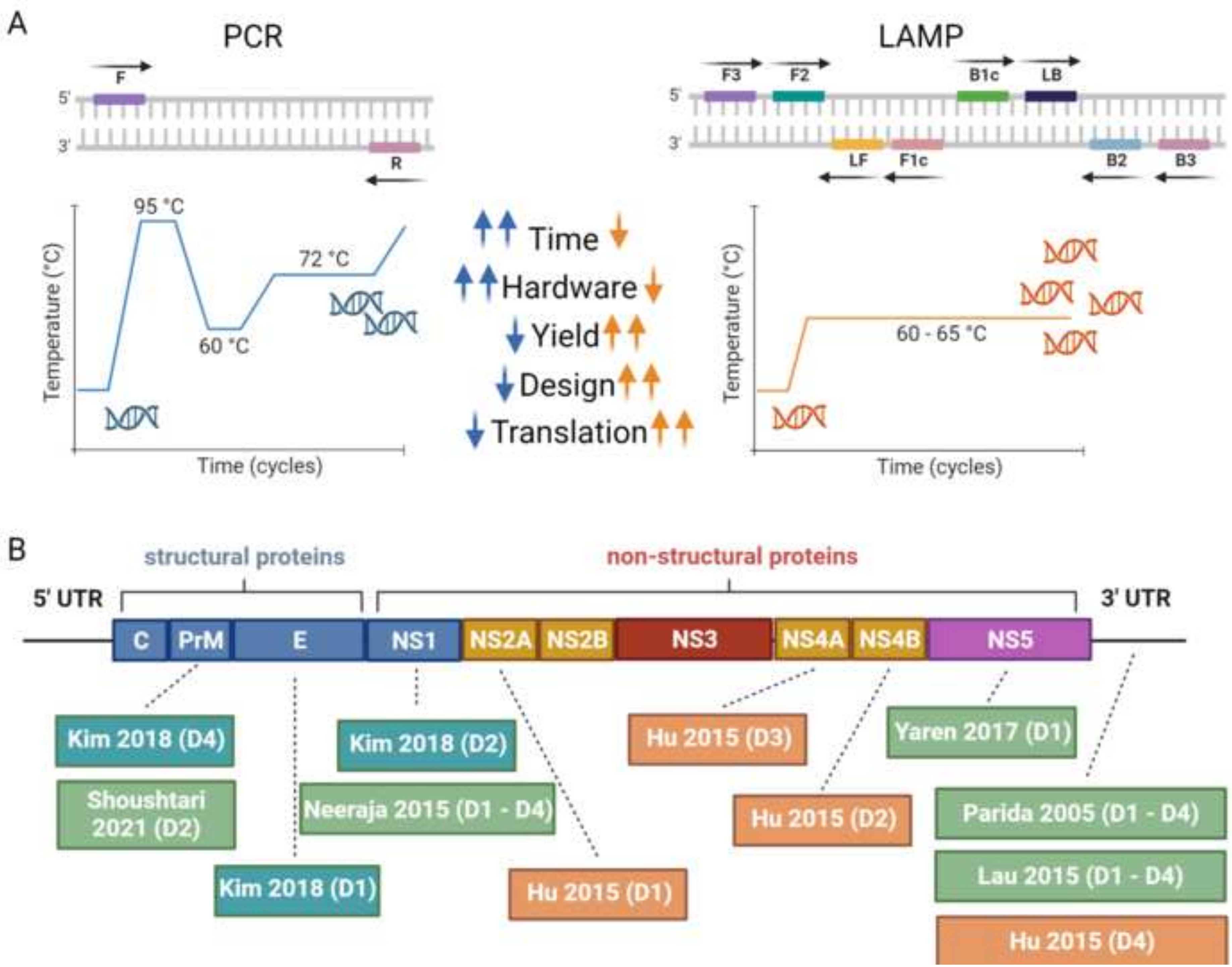

S1 Table - PRISMA-ScR checklist

Click here to access/download
Supporting Information
PRISMA-ScR checklist.docx

Click here to access/download
Supporting Information
S1 Data v2.xlsx

Analytical and diagnostic performance characteristics of reverse-transcriptase loop-mediated isothermal amplification assays for dengue virus serotypes 1-4: a scoping review to inform potential use in portable molecular diagnostic devices

Paul Arkell[1,]*, Dumrong Mairiang[2,3], Adisak Songjaeng[2,4], Kenny Malpartida-Cardenas[1,51], Kerri Hill-Cawthorne[1], Panisadee Avirutnan[2,3,4], Pantelis Georgiou[1,56], Alison Holmes[1,6], Jesus Rodrigquez-Manzano[1,5]

1. *Centre for Antimicrobial Optimisation, Department of Infectious Disease, Imperial College London, Hammersmith Hospital, Du Cane Road, London, W12 0NN. United Kingdom.*
~~1.~~
2. *Siriraj Center of Research Excellence in Dengue and Emerging Pathogens, Faculty of Medicine Siriraj Hospital, Mahidol University, Bangkok-noi, Bangkok 10700, Thailand.*
3. *Molecular Biology of Dengue and Flaviviruses Research Team, Medical Molecular Biotechnology Research Group, National Center for Genetic Engineering and Biotechnology (BIOTEC), National Science and Technology Development Agency (NSTDA), Bangkok 12120, Thailand.*
4. *Division of Dengue Hemorrhagic Fever Research, Department of Research and Development, Adulyadet Vikrom Building, 12th floor, Faculty of Medicine Siriraj Hospital, Mahidol University, Bangkok-noi, Bangkok 10700, Thailand.*
5. *Department of Electrical and Electronic Engineering, Imperial College London, Exhibition Road, SW7 2AZ. United Kingdom.*
6. *David Price Evans Global Health and Infectious Disease Research Group, University of Liverpool*

*** Corresponding Author:**
Dr Paul Arkell, Centre for Antimicrobial Optimisation, Imperial College London, Hammersmith Hospital, Du Cane Road, London.W12 0NN. United Kingdom.
Email: p.arkell@imperial.ac.uk

Keywords: Diagnosis, dengue, molecular detection, LAMP, loop-mediated isothermal amplification, serotypes, review

ABSTRACT

Background: Dengue is a mosquito-borne disease caused by dengue virus (DENV) serotypes 1-4 which affects 100-400 million adults and children each year.~~–~~ Reverse-transcriptase (RT) quantitative polymerase chain reaction (qPCR) assays are the ~~modern~~current gold-standard in diagnosis and serotyping of infections, but their use in low-middle income countries (LMICs) has been limited due to ~~significant~~ laboratory infrastructure requirements. Loop-mediated isothermal amplification (LAMP) assays do not require thermocycling equipment and therefore have the potential to be deployed outside laboratories and/or miniaturised.~~–~~ This scoping literature review aimed to describe the analytical and diagnostic performance characteristics of ~~all~~ previously developed serotype-specific dengue RT-LAMP assays and evaluate potential for use in ~~novel~~ portable molecular diagnostic devices.

~~-~~**Methods:** A literature search in Medline was conducted.~~–~~ Studies were included if they were listed before 4th May 2022 (no prior time limit set) and described the development of any serotype-specific DENV RT-LAMP assay (‘~~studies describing~~ original assays’) or described the further evaluation, adaption~~evaluated~~ or ~~/~~implementation~~ed~~ ~~one~~ of these assays ~~(‘studies using previously described assays’)~~.~~–~~ Technical features ~~(nucleic acid extraction, reaction mix, primer sequences, detection methods)~~, analytical and diagnostic performance characteristics ~~(lower-limit of detection (LLOD), linear range, analytical specificity), diagnostic performance characteristics (diagnostic sensitivity and specificity) and details of further usage (modifications, use in novel/prototype molecular diagnostic devices)~~ were collected for each assay.

Results: Eight studies describing original RT-LAMP assays for dengue serotyping were identified. These ~~. These~~ were heterogenous in design and reporting~~, with some omitting key experimental details~~.~~–~~ Assays’ lower ~~When described,~~ limit of detection (~~L~~LLOD)~~s~~ and linear range~~s~~ of quantification were comparable to RT-qPCR (with lowest reported values $2.2x10^1$ and $1.98x10^2$ copies/ml, respectively, for studies which quantified target RNA copies) and a~~. A~~nalytical specificity

was was also high, though the detail and number of interference experiments conducted was often omitted and non-specificity was a cited reason for multiple subsequent assay modifications. – When evaluated, diagnostic sensitivity and specificity for many assays wereperformance was also high, though various reference gold-standard assays or clinical diagnostic criteria were used as reference testsvaried widely, prohibiting comparison between assays. – Fourteen studies using previously described assays were identified, including those where samples were tested directly (i.e. without nucleic acid extraction), reagents were lyophilised or 'printed' into microfluidic channels, and where several novel detection methods were used (including the use of smart phone cameras, electrochemical sensing, and MinION sequencing).

Discussion: – Serotype-specific DENV RT-LAMP assays have potential to be use inare high-performing and have potential to be used in portable molecular diagnostic devices for diagnosing and serotyping dengue infections if they can be coupled with miniaturisedintegrated with sample extraction and detection methods. – SStandardised assay evaluation including rigorous efforts to assure analytical specificity and more detailed reporting of assay validation studiesperformance characteristics in general would be beneficial.

Formatted: Line spacing: Double

BACKGROUND:

Dengue is a mosquito-borne disease caused by dengue virus serotypes 1-4 (DENV 1-4). International travel, urbanisation and climate change have contributed to increasing global incidence with more thanup to half the world's population across 125 tropical and sub-tropical countries now at-risk. (1–3) There are an estimated 100-400 million infections annually which cause a spectrum of disease ranging from asymptomatic or mild, self-limiting symptoms to severe forms of the disease, including dengue haemorrhagic fever and dengue shock syndrome.(4,5) – 'Secondary infection', when an individual is infected for a second (or subsequent) time in their life by a different serotype to their 'primary infection' is most likely to result in severe disease.(6–8)

After recovery from a first dengue infection ('primary infection') there is typically long-lasting immunity which protects against subsequent infection with the same DENV serotype.[6] However, due to immunological phenomena including 'antibody-dependant enhancement' (ADE), those who are subsequently infected with a different serotype ('secondary infection') are at significantly increased risk of DHF and DSS.[7,8] Therefore, the shift of theshifts in the predominant circulating DENV serotype in a geographical area is frequently can be associated with more severe disease outbreaks.(9) Better access to serotype-specific diagnostic testing for dengue would may improve case-management, surveillance and disease control.(10)

The dengue diagnostic gap

Diagnosis of dengue infection can be made based on clinical features but should be confirmed using a diagnostic test wherever possible.[11] Traditional techniques for confirming dengue include virus isolation by cell culture, detection of host antibodies and/or virus antigens and lateral flow assays (LFAs). Virus isolation by cell culture may produce definitive serotype-specific evidence of DENV infection but requires skilled technicians and takes several days to generate results[12]. Detection of host antibodies and/or virus antigens using laboratory-based serological assays may differentiate primary from secondary infection but can lack sensitivity and cross-react with responses to other

~~flaviviruses[13,14]. Finally, LFAs are portable, do not require specific training, can be produced at low cost, but have variable sensitivity and do not differentiate the infecting serotype[15].~~

Reverse-transcriptase polymerase chain reaction (RT-~~q~~PCR) assays are the ~~modern~~current gold-standard in diagnosis and serotyping of dengue infections.(11) These ~~have been developed to~~ detect DENV ribonucleic acid (RNA) ~~presenting~~ which is present in clinical samples from the ~~time of symptom-onset~~onset of symptoms in both primary and secondary ~~dengue~~infection. ~~RT-PCR assays use a~~A ~~reverse-transcriptase~~RT enzyme is used to synthesise complementary deoxyribonucleic acid (cDNA) from a target RNA sequence, and a ~~*Taq*~~ DNA polymerase enzyme is used to amplify the cDNA. ~~RT-q~~PCR primers can be designed to either bind regions of the DENV genome which are conserved across serotypes (resulting in a 'generic' dengue assay), or regions which are specific to an individual serotype (resulting in a serotype-specific assay).(12) ~~Conventional RT-PCR assays require a separate step (subsequent to nucleic acid amplification) in which the product is detected. Quantitative (or 'real-time') RT-PCR assays (RT-qPCR assays) use an intercalating dye or a hydrolysis-based probe such that nucleic acid is amplified and detected simultaneously.~~ ~~RT-q~~PCR assays can quantify DENV RNA if performed in real-time PCR (RT-qPCR), and ~~and may~~can be multiplexed with assays for other pathogens. (13,14) ~~RT-qPCR is highly sensitive and specific and are therefore the gold-standard technique in diagnosis of dengue.[11]~~ However, in all PCR assays, amplification of nucleic acid occurs during repeated cycles of heating and cooling ~~(~~to achieve precise denaturation and annealing temperatures~~)~~, requiring significant laboratory infrastructure and a reliable power supply. ~~It also requires~~They also require skilled operators and systems for quality and safety. As such, deploying PCR facility in many low-middle income countries (LMICs) is impractical. Patients in most settings do not receive serotype-specific DENV testing ~~and consequently there cannot be effective disease surveillance which provides early warning of serotype-switching~~and this may impact disease surveillance and control efforts. A multicentre observational study on the global availability of testing ~~has~~ highlighted ~~a major gap~~the diagnostic gap in ~~diagnostic availability in~~

LMICs, which particularly affects ~~primary care, and includes diagnostic tests for infectious disease~~remote and regional areas.(15,16)

Loop-mediated isothermal amplification (LAMP) as a potential solution

Loop-mediated isothermal amplification (LAMP) is an isothermal nucleic acid amplification method first described in 2000 by Notomi et al.(17) When coupled with a reverse-transcriptase step (RT-LAMP), it can be used to detect ~~pathogen~~ RNA (Figure 2). Samples can be analysed directly, but usually undergo a nucleic acid extraction step to isolate and purify RNA, removing undesired components which may inhibit or otherwise affect the efficiency of the amplification reaction. LAMP uses a *Bacillus stearothermophilus* (*Bst*) DNA polymerase enzyme which possesses high autocycling strand displacement activity, allowing DNA synthesis to occur at a constant temperature (typically between 60-65 degrees Celsius). Multiple primers are used to create continuous loop structures during amplification. Primers ~~, which~~ may be designed to produce either generic or serotype-specific dengue assays. ~~These~~ LAMP primers include forward and backward outer primers (F3 and B3, respectively), forward and backward inner primers (FIP and BIP), and forward and backward loop primers (FLP and BLP, which are not essential but can improve efficiency of the reaction). Amplified nucleic acids can be detected and/or quantified using various methods including visual inspection, turbidometry, gel electrophoresis, real-time monitoring using intercalating dyes and hybridisation with fluorescent probes, or non-fluorescence methods such electrochemical sensors.(18–21)

Because LAMP assays do not require thermocycling equipment, they have long been considered potentially more suited to miniaturisation and/or deployment outside ~~specialist centres~~the laboratory setting than PCR assays, including for dengue and other neglected tropical diseases. (22,23)

Evaluating RT-LAMP assay performance

Studies which evaluate RT-LAMP assays may include measurement of various analytical performance characteristics (i.e. those which are inherent to the assay) and/or diagnostic (clinical) performance characteristics (i.e. those which become relevant when the assay is used to detect a condition or disease).(24–26)

Analytical performance characteristics include lower limit of detection (LLOD, also known as 'analytical sensitivity' which is ~~a measure of the ability of the assay to detect very low~~defined as the lowest concentration ~~s~~ of a given substance that can be detected) and the linear range of quantification (also known as the 'reportable range', which for quantitative assays is the span of test result values over which the accuracy of the measurement can be verified). Additionally, analytical specificity (which is the ability of an assay to detect only the intended target and the absence of 'cross-reaction' with potentially interfering nucleic acids or specimen-related conditions) can be determined by interference studies which test 'no template controls' and/or samples containing potentially interfering substances or non-targeted biomarkers. If/when amplification does occur, various techniques can be used to verify authenticity of the DNA product, and hence analytical specificity of the assay.~~–~~ These include assessment of amplicon ~~size~~specificity ~~digestion~~ using a specific restriction enzyme and agarose gel electrophoresis, amplification and ~~(so that the amplicon size can be compared to what would be expected from the target sequence),~~ melting curve studies ~~(which assess DNA denaturation temperature and compare to what would be expected)~~, and nucleic acid sequencing techniques.(27,28) Assuring analytical specificity is particularly important ~~for LAMP~~in assay design ~~assays~~ because several phenomena including the formation of amplifiable primer-dimers and hairpin structures can lead to false-positivity.(29–31)

Diagnostic performance characteristics include diagnostic sensitivity (which is the ability of a test to correctly classify an individual as having a condition or disease, i.e. the number of true positive results as a fraction of the total number individuals with the condition or disease) and diagnostic specificity (which is the ability of a test to correctly classify an individual as not having a condition or

disease, i.e. the number of true negative results as a fraction of the total number of individuals without the condition or disease). An important consideration for diagnostic accuracy studies is the choice of reference standard, which is used to compare the performance of the 'index test' against. This may be an alternative 'gold-standard' assay or may be based on validated clinical diagnostic criteria.(24)

~~If RT-LAMP assays are high-performing (i.e. comparable to RT-qPCR assays), they could be used in a new generation of portable molecular diagnostics for dengue.~~

Aim

The primary aim of this scoping review was to describe the technical features and ~~analytical and diagnostic~~ performance characteristics of ~~all~~ previously developed serotype-specific dengue RT-LAMP assays. ~~-~~ The secondary aim~~s were~~was to ~~, characterise the their current application of assays in in entomological, epidemiological or and clinical studies of dengue, and~~ evaluate their potential for use in ~~novel high-performing~~ portable molecular diagnostic devices.

METHODS

Methods for this scoping literature review were developed ~~according~~ with reference to the Preferred Reporting Items for Systematic reviews and Meta-Analyses extension for Scoping Reviews guideline.(32)~~-~~ A literature search in Medline was conducted on 4th May 2022. The search strategy was constructed by two investigators from combinations of medical subject headings (MeSH) and keywords (~~see~~ Table 1). Results were imported into Covidence software and deduplicated.(33) ~~PA and DM examined c~~Citations were reviewed at a title/abstract level for potential inclusion, then ~~examined each study~~ at ~~a~~ full text level for inclusion by two authors. Discrepancies were resolved by discussion until consensus was reached. Citation lists from all studies examined at full text level, as well as those from all review articles identified by the original search, were also reviewed.

Studies were included if they were listed before 4th May 2022 (no prior time limit set) and fulfilled either of the following criteria: 1) Described one or more newly developed RT-LAMP assay which was designed to detect a single DENV serotype 1-4 ('studies describing original assays'); 2) Described the further evaluation, adaption and/or implementation of one of the aforementioned original RT-LAMPthese assays ('studies using previously described assays'). Assays were considered the same if they included the same sets of nucleic acid primers, even if other features of the assay differed (for example the method of nucleic acid extraction or detection of the amplified product). Studies were excluded if they did not detail the primer sets which were used (either within the publication, supplementary material or by reference), if they described only generic dengue assays, or if they were not written in English language. All types of laboratory or clinical study design were eligible.

Data were collected from included studies according to a pre-determined proforma. This was designed based on the required performance characteristics which are needed before implementation of laboratory-developed tests, as detailed in the Revised Clinical Laboratory Improvement Amendments (CLIA) of 2003(34) (analytical performance characteristics) and the updated Standards for Reporting Diagnostic Accuracy (STARD) statement of 2015 (diagnostic performance characteristics).(35) Where LLOD was given in plaque forming units (PFU) or RNA copies per microlitre, this was converted to PFU or RNA copies per reaction (by multiplying by the reported reaction volume). Technical features of the assay (extraction method, reaction mixture ingredients, primer sequences, incubation temperature and detection method) were also collected.

RESULTS

Study characteristics

The database search retrieved 87 unique articles, of which 46 were excluded based on titles and abstracts. Two articles requiring full text review were also identified through review of citations.

Forty-three full texts were therefore assessed for eligibility, of which 22 were included and 21 were excluded. The commonest reason for exclusion at full text review was ‘assay was generic (i.e. not a serotype-specific assay)’, which applied to 12 articles. Of the 22 studies included, 8 described original assays and 14 described the further evaluation, adaption, or implementation of a previously developed assay~~14 used previously described assays~~. A ~~consort~~ study workflow diagram is shown in Figure 1.

Technical features of original RT-LAMP assays

Eight sets of o~~O~~riginal RT-LAMP assays were developed by Parida et al (2005), Neeraja et al (2015), Hu et al (2015), Lau et al (2015), Yaren et al (2017), Kim et al (2018), Lopez-Jimena et al (2018) and Shoushtari et al (2021). (36–43)– They were designed by obtaining sequences for dengue virus serotypes 1-4 from GenBank/NCBI database (7/8) or other sources (1/8). Various methods and software packages were used to identify potential template regions where sequences were conserved within sequences from the same serotype (but distinct from other serotypes and organisms) ~~+/-~~ and ~~further~~ assess possible secondary structures of primers ~~for dimerization and/or other adverse features~~. This included DNASIS software (Hitachi, Japan, 2/8 studies), Primer-Explorer V5 (Eiken Chemical Co. LTD, Tokyo, Japan, 2/8), LAMP designer (Primer biosoft, America, 1/8), Primer-Explorer V3 (Eiken Chemical Co. LTD, Tokyo, Japan, 1/8), OligArch v2 (FfAME, Alachua, FL, 1/8), PrimerCompare v1 (FfAME, Alachua, FL, 1/8), and various R packages (1/8). Figure 2 shows a schematic of the dengue genome and the position of primer-binding for each serotype-specific RT-LAMP assay.

Out of the eight original assays, seven performed nucleic acid extraction using commercially available kits (most commonly QIAamp viral RNA mini kit, QIAGEN, Hilden Germany). ~~In one case the extraction method was not clear.~~ All assays used commercially available preparations of *Bst* DNA polymerase and a reverse transcriptase enzyme (Avian Myeloblastosis Virus, AMV, or RTx reverse transcriptase~~s~~). Some assays used commercially available LAMP reaction mixes, while others used

bespoke mixes which included deoxynucleoside triphosphates, betaine, Tween 20, $(NH4)_2SO4$, $MgSO_4$ (or $MgCl_2$), KCl and Tris–HCl. Details of the extraction method(s), enzymes reaction mixtures and primer sequences for each assay are included in appendix 1.

Analytical and diagnostic performance of original RT-LAMP assays

When determined in 7/8 studies, LLOD of assays was between $2.5x10^{-3}$ and $1.22x10^{0}$ plaque-forming unit (PFU)/reaction (for studies which quantified target in PFU) and between $2.2x10^{1}$ and $8.25x10^{2}$ copies/reaction (for studies which quantified target in RNA copies). However, some studies did not give detailed description of the method for quantification of viral particles and/or copies of template RNA used in LLOD experiments. Furthermore, it was sometimes not clear whether the cited concentrations referred to those of original samples, the elution buffer after nucleic acid extraction (i.e. the extract), or the final reaction mix. In this review, concentrations have been converted to 'per-reaction values', as best possible from the information available in manuscripts. When determined in 3/8 studies, the linear range of quantification went as low as $2.5x10^{0}$ PFU/reaction (for the study which quantified target in PFU) and as low as $1.98x10^{2}$ copies/reaction (for studies which quantified target in RNA copies), but similar difficulties interpreting quantification methods and cited concentrations were encountered.

Analytical specificity was usually assessed by testing viral particles of genetically similar viruses (including other flaviviruses and the otherdiscordant DENV serotypes and other flaviviruses), or their synthetic RNA or DNA templates. – , with virtually Virtually no incidents of non-specific amplification being were reported across all studies. However, the total number of runs reactions which including potentially interfering substances (or 'no template controls') conducted during these interference studies was often not reported, (or was fewer than 10). Authenticity of the amplified product was reportedly evaluated using digestion withusing a specific restriction enzyme and agarose gel electrophoresis in 3/8 studies, nucleic acid sequencing in 3/8 studies, melting curve studies in 1/8 study, and was not done in 1/8 studyies. However, sometimes these data were not presented in

results, and it was rarely clear whether authentication was undertaken for all experiments (i.e. every sample which was determined positive), or only a subset.

Diagnostic performance of original RT-LAMP assays

Assessment of diagnostic performance occurred in 6/8 studies. This usually involvedMost often a panel of 'positive samples' which were parallel-twere used, which had been estedtested in parallel using alternative methods (5/6 studies), and/oror which had been characterised previously and assigned 'dengue positive' based on clinical criteria (1/6 studies). – A , and a panel of 'negative samples' from healthy individuals were also used Iin most studies (5/6). Studies usually used RT-qPCR was most often used as as a reference assaystandard, but some studies did not clearly detail which assay (or clinical case definition, or composite) whatwhich test and/or clinical case definition was being used as a reference standard. In 1/61/6 study, significantly higher positivity from RT-LAMP assays testing resulted in significantly higher positivity than RT-qPCR, was observed, compared to RT-qPCRwhich . This was interpreted as superior diagnostic sensitivity of RT-LAMP but may also have been due to low diagnostic specificity.

Adaption of original RT-LAMP assays towards portable molecular diagnostic devices

Fourteen **studies** described the further evaluation, adaption, or implementation of a previously developed assay. – None of these studies present a working portable molecular diagnostic device for analysis of individual samples which has been deployed and thoroughly evaluated outside the laboratory setting. – However, the following technological advances were presented:

Narrative summary of included studies

Technical features, analytical and diagnostic performance characteristics of original assays (8 studies) and key modifications when they were used subsequently (14 studies) are summarised in Table 2 and detailed below.

<Table 2 here>

Parida et al (2005)[38]

In this study RT-LAMP assays for each of DENV1-4 serotypes were developed. Primers were designed based on 3' non-coding regions (NCRs) of each DENV genome using DNASIS software (Hitachi, Japan). Incubation occurred at 63 degrees Celsius for 60 minutes, though for unstated reasons there was determination of positivity at 30 minutes. There was real time monitoring of the reaction using turbidometry as well as visual inspection of the product with and without the addition of a nucleic acid stain (SYBR Green I). LLOD for the DENV1 assay was 1 plaque-forming unit (PFU)/ml (=$2.5x10^{-2}$ PFU/reaction) and for the DEN2-4 assays was 0.1 PFU/ml (=$2.5x10^{-3}$ PFU/reaction). The linear range was $1x10^{2}$ – $1x10^{6}$ PFU/ml (= $2.5x10^{0}$ – $2.5x10^{4}$ PFU/reaction) for the DENV1 assay and $1x10^{1}$ – $1x10^{6}$ PFU/ml (=$2.5x10^{-1}$ - $2.5x10^{4}$ PFU/reaction) for the DENV2-4 assays. Analytical specificity was verified by testing Japanese encephalitis virus (JEV), West Nile virus (WNV) and St Louis encephalitis virus nucleic acid templates which did not amplify. Authenticity of the amplified products were established by digestion using a specific restriction enzyme and agarose gel electrophoresis, and by sequencing. Diagnostic performance was assessed using 25 serum samples from confirmed cases of dengue, 38 suspected cases of dengue and 20 healthy individuals, with positivity by conventional RT-PCR and/or virus isolation being considered the reference standard. Serotype-specific results were not given but it appears that overall diagnostic sensitivity and specificity of all four assays together were both 100%.

The Parida et al assays were used in six subsequent studies. In 2011 Li et al adapted the assay, performing optical RT-LAMP detection with a fluorescent detection reagent (Eiken Chemical Co. Ltd, Tokyo, Japan) and real-time monitoring with SYBR Green I (Qiagen).[39] Positivity was determined at a reduced timepoint (20 minutes) because non-specific reactivity to blank controls was observed beyond 30 minutes. The assays detected 52/52 (100%) cultured DENV strains with no cross-reactivity from other viruses. When applied to serum samples from patients who reportedly had DENV1 or

Formatted: Font: Not Bold

DENV3 infection (case definition not stated), the assays were positive in more instances than a one-step RT-PCR assay performed in parallel. These results were interpreted to show high clinical sensitivity of the RT-LAMP assays rather than a lack of clinical specificity. The assay was also applied to 'blood sucked female Aedes sp. mosquitoes' and was positive in 4 instances. In 2012 Lo et al used the DENV2 assay to demonstrate that amplified RT-LAMP products can be detected using a colorimetric assay on wax-patterned paper.[40] In 2013 Chagan-Yasutan et al used the assays to determine infecting dengue serotype in individuals enrolled in an observational study which examined biomarkers in acute dengue infection.[41] In 2017 Yamagishi et al (2017) used the assays with a 60 to 90 minute incubation adapted the Parida et al assay, analysing samples directly (i.e. without NAnucleic acid extraction) and loading at either 63 or 65 degrees Celsius (discrepant protocols are presented in the manuscript).[42] aAmplified products were purified using a magnetic bead procedure and then loaded into a portable MinION sequencer to , with individual and consensus sequences being mapped to reference genomes to determine the serotype. Initially an entire MinION flow cell was used for each sample, then changes to the LAMP primers were made such that amplicons were barcoded and could be combined and run together (to scale the assays). The authors Sequencing was deemed necessary because erroneous LAMP signals were observed from negative control sampleshighlight the necessity for sequencing analysis after LAMP and cite erroneous signals including from samples they analysed from healthy individuals, and the. workflow Various evaluations of assay performance were presented, and it was ultimately was ultimately used under 'field conditions' at a small clinic in Indonesia. In this assay samples were analysed directly.(44)

In 2021 Kumar et al adapted one of the assays (serotype not stated) by coating primers with either biotin or digoxigenin, precipitation of the amplified product using polyethylene glycol, and inducing clumping with streptavidin- or anti-digoxigenin-coated magnetic particles.[43] This produced a 'magnetic' assay which could be multiplexed with one for chikungunya and visualised with the naked eye. In 2022 Gaber et al used the assays in a retrospective study of 51 serum samples from patients

admitted with suspected dengue infection in Egypt.[44] Positivity was determined after a prolonged incubation of 60 minutes using naked eye visualisation and agarose gel electrophoresis. In 31/51 (60.8%) samples at least one dengue serotype was detected, and 'mixed infection' with more than one serotype was observed in 29/31 (83.9%) of these. Thirty-six pools of macerated mosquitos were also tested with high positivity. These results were interpreted to show high clinical sensitivity of the RT-LAMP assays rather than a lack of clinical specificity.

Neeraja et al (2015)[45]

Formatted: Font: Not Bold

In this study RT-LAMP assay for each of DENV1-4 serotypes were developed. Primers were designed based on the non-structural protein 1 (NS1) regions of each DENV genome using DNASIS software (Hitachi, Japan). Incubation occurred at 63 degrees Celsius for 35 minutes, which was followed by a heating and cooling step to stop the reaction. There was visual inspection of the product with and without the addition of SYBR Green I. The LLOD and linear range of the assay were not reported. Analytical specificity was verified by testing samples which were positive for other flaviviruses (sic) including JEV, WNV, hepatitis C virus (HCV) and chikungunya virus (CHIKV), which did not amplify. Authenticity of the amplified products were established using digestion using a specific restriction enzyme and agarose gel electrophoresis, and by sequencing (with 100% sequence homology reported). Diagnostic performance was assessed using 250 serum or plasma samples from individuals suspected clinically of having dengue and which were positive for other markers of dengue infection (NS1 antigen, anti-dengue IgM, dengue RT-qPCR) and 50 healthy individuals. Various analyses using different reference standards were reported, including 100% sensitivity and 95.0% specificity against RT-qPCR, respectively.

The Neeraja et al assays were used in one subsequent study. Dave et al examined ocular tissue in a series of three cases of panopthalmitis associated with acute dengue infection.[46] There was positivity in all three cases but serotypes were not reported.

Formatted: Font: Not Bold

Hu et al (2015)[47]

Formatted: Font: Not Bold

In this study RT-LAMP assay for each of DENV1-4 serotypes were developed. DNASIS software (Hitachi, Japan) was used to identify potential target regions of each DENV genome which had high sequence variability across DENV1-4 and then LAMP designer (Primer biosoft, USA) was then used to design primer sequences. Incubation occurred at 63 degrees Celsius for 45 minutes. There was real time monitoring of the reaction and visual detection, both using SYBR Green I as a source of fluorescence. LLODs for all four assays were $1x10^1$ copies/uL (=$2.5x10^2$ copies/reaction) and linear ranges were $1x10^1$ – $1x10^7$ copies/uL (=$2.5x10^2$ – $2.5x10^8$ copies/reaction), respectively. Analytical specificity was verified by testing JEV, yellow fever virus (YFV), herpes simplex virus and Epstein-Barr virus 10 times, which did not amplify. Authenticity of amplified products appeared to have been established by digestion using a specific restriction enzyme and agarose gel electrophoresis, and reportedly also by sequencing. Diagnostic performance was assessed using 190 serum samples from patients who were 'confirmed to be infected [with] dengue by clinical diagnosis', as well as 20 healthy individuals. A reference standard was not clearly defined, but positivity of these samples by a RT-PCR assay was significantly lower than by RT-LAMP. These results were interpreted to show high clinical sensitivity of the RT-LAMP assays (reported as 97.5%-100%) rather than a lack of clinical specificity (reported as 100%).

The Hu et al assays were used in one subsequent study. Ganguli et al (2017) used the DENV1 and DENV3 assays. Each primer set (along with those for ZIKV and CHIKV) were printed printed and dried and dried Hu et al primers onto microfluidic channels in bespoke sample-processing and amplification chips. Samples of whole blood spiked with ZIKV or extracted DENV1, DENV3 or CHIKV RNA were processed. Amplification occurred on the chip while it is housed within a 3d-printed light-proof cradle and a smart phone was used to perform real time detection of fluorescence in each channel. (45)While LLOD was not presented for DENV1 or DENV3, it was 1.56x10^5 PFU per ml of starting blood for ZIKV.

Lau et al (2015)[49]

~~In this study RT-LAMP assay for each of DENV1-4 serotypes were developed. Outer and inner primers were designed based on the 3' NCRs of each DENV genome using Primer-Explorer V3 software (Eiken Chemical Co. LTD, Tokyo, Japan), and loop primers were designed manually. Incubation was at 65 degrees Celsius. There was real time monitoring of the reaction using turbidometry as well as visual inspection of the product with HNB dye (Sigma, USA). Positivity was determined at 30 minutes for DENV1-3 assays, and at 45 minutes for the DENV4 assay. LLOD was 'as low as 10 copies', though the exact units of measurement are not clear. A linear range was not reported. Analytical specificity was verified by testing other viruses including JEV, which did not amplify. Authenticities of amplified products were not established. Diagnostic performance was assessed using 189 serum samples from patients with suspected dengue and 24 serum samples from healthy donors. The reference standard was a composite of three tests, including RT-qPCR (ran in parallel), ELISA (ran in parallel, but the antibody class being detected was not stated) and RT-LAMP (which was also the index test). Clinical sensitivity and specificity were both found to be 100%.~~

~~The Lau assays have been used in four subsequent studies.~~ Minero et al (2017) ~~amplified synthetic DENV2 DNA and used~~ developed and applied two different detection methods using optomagnetic spectroscopy to the Lau et al assays.~~[50]~~ First, the interaction between biotinylated FIP or LF primers (which incorporate into amplicons during the LAMP reaction) and streptavidin-coated magnetic nanoparticles (included in the reaction mixture) was observed in real-time. Second, a method was devised to try and discriminate between 'true positive' and 'spurious' LAMP amplicons using a ~~. A~~ 3'-biotiylated 'loop-validating' DNA probe.~~–~~ In this study t~~was designed to target one of the emerging loops of the LAMP amplicon, and a 'loop-validating' probe was used instead of the LF primer. During melting curve analysis over a temperature ramp, significant differences in optomagnetic spectra of true positive vs. spurious amplicons were observed. T~~he authors highlight the common problem of spurious amplicons in LAMP assays and the importance of having a readout method which is not prone to detection of these. (46)

Priye et al (2017) ~~used the DENV1-4 primers in a single tube to amplify and detect DENV1 using an intercalating dye (SYTO 9) for detection.[51] They also describe~~described a ‘quenching of unincorporated amplification signal reporters’ (QUASR) technique which ~~is~~ was used to multiplex the Lau et al ~~the~~ DENV1 and DENV2 assays. ~~(performing serotype-specific detection) as well as assays for ZIKV and CHIKV. Reactions occurred directly. The QUASR technique involved labelling the~~ The BIP primer was labelled with cyanine-5 ~~(or other label)~~ and ~~including~~ a short complimentary quenching probe was included, resulting in ~~. This resulted in~~ fluorescence upon cooling ~~of the reaction~~ if specific amplification had occurred. The whole workflow was transferred into a ‘smart phone-operated LAMP box’ which included a heating module, an assay reaction housing module and an optical-detection/image-analysis module and gave a qualitative ~~(binary)~~ result for each target. ~~LLOD for the zika virus assay of 10 PFU/ml of unextracted sample was reported.~~(47) ~~Meagher et al (from the same group) used the assays to demonstrate a LAMP phenomenon of non-specificity which they term ‘rising baseline’ (considered separate from ‘exponential amplification with no template’, which can also occur).[31] They modified DENV2 and DENV4 primer sets to reduce hairpin formation and/or primer-dimer interaction and observed that the modified assays had low rate of spontaneous ‘false positive’ amplification, even with extended incubations. Sigera et al performed a clinical evaluation of the assays.[52] Plasma samples from 122 patients with suspected dengue infection were and then amplified using all four primer sets combined into a single reaction mixture, and monitored in real-time by turbidometry and visual inspection. Samples were tested in duplicate but interpretation of any discrepant results was not stated. Compared to a RT-PCR assay (reference standard), diagnostic sensitivity was 73.8%, diagnostic specificity was 95.2%, PPV was 96.7% and NPV was 65.6%.~~

~~**Yaren et al (2017)**[53]~~

~~In this study a RT-LAMP assay for DENV1 was developed. Potential primer sets were designed using in-house software (OligArch v2, FfAME, Alachua, FL) and compared using PrimerCompare v1 (FfAME, Alachua, FL). Additionally, fluorescence-labelled (TAMARA) LB strand-displaceable probes and~~

quencher probes were included so that the assay could be multiplexed with other targets. Incubation occurred at 65 degrees Celsius for 60-90 minutes. Fluorescence was initially detected in real-time, then by a smart phone with an orange filter. Analytical performance experiments determined the LLOD for the assay to be 1.22 PFUs per reaction, when used in single-plex format. A linear range was not reported. In multiplex format, no cross-reactivity was observed between the DENV1 assay and RNA from either ZIKV or CHIKV. Authenticities of amplified products were not established. In one format of the assay, lyophilised reagents were used. Diagnostic performance was not assessed.

The Yaren assay was used in one subsequent publication by the same group which was a protocol manuscript without new results.[54]

Kim et al (2018) [55]

In this study RT-LAMP assays for DENV1, DENV2 and DENV4 serotypes were developed. F3, B3, FIP, BIP primers were designed using Primer-Explorer V5 (Eiken Chemical Co. LTD, Tokyo, Japan). FLP and BLP primers were not included. Incubation occurred for 40 minutes at between 65.0 and 69.7 degrees Celsius. LLODs were between 3.55 and 33copies/uL (=between 8.88x10^1 and 8.25x10^2 copies/reaction), and linear ranges went as low as 7.91 copies/uL (1.98x10^2 copies/reaction). Analytical specificity was verified by testing RNAs of DENV3, norovirus, rotavirus and bovine viral diarrhoea, which did not amplify. The authenticities of amplified products were not established. Diagnostic performance of the assays were not assessed and no subsequent publications using the assays were identified.

Lopez-Jimena et al (2018)[56]

In this study RT-LAMP assay for each of DENV1-4 serotypes were developed. Primers were designed using various R packages and then a modified version of LAVA.[57] Incubation occurred at 64 degrees Celsius for 45 minutes (DENV1), 90 minutes (DENV2), 75 minutes (DENV3) or 50 minutes (DENV4).

There was real-time detection of a fluorochrome dye. LLOD was determined by probit analysis to be 22, 542, 92 and 197 copies per reaction for each assay, respectively. Linear ranges were not reported. Analytical specificity was verified by testing several RNA viruses and DNA pathogens, which did not amplify. Authenticity of amplified products was established by melting curve analysis, with a single peak observed for each assay. Diagnostic performance of the assays was assessed using 31 blood samples, 11 serum samples and 36 RNA extracts from patients in various dengue-endemic countries, with an RT-qPCR assay being used as a reference standard. Results from several analyses are presented including one where false-negative samples were identified and then re-analysed. Diagnostic sensitivities and diagnostic specificities were between 70.8-100% and 50-100%, respectively.

The Lopez-Jimena assays were used in one subsequent publication. Hin et al (2021) used The Lopez-Jimena assays all primer sets (together with primers for various other viral, bacterial and parasitic infections) in an 'FeverDisk'.[58] This automated device performings sample lysis, nucleic acid extraction, combination with lyophilised reagents and aliquoting into reaction chambers. Up and up to to 12 parallel LAMP reactions can occur andwhich are detected in real-time using fluorescence (the 'FeverDisk'). Analytical performance characteristics for the DENV assays in this format were not determined. However, but specimens from some participants in Senegal and Sudan were tested, with three samples being were tested positivepositive.(48) (all of which were also positive by reference methods).

Kumar et al (2022) coated Prida et al primers with either biotin or digoxigenin, precipitated the amplified product using polyethylene glycol, and induced clumping with streptavidin- or anti-digoxigenin-coated magnetic particles. This produced a 'magnetic' assay which could be interpreted visually and multiplexed with another assay.(49)

Table 2 and S1 Data summarise thes included studies included in this review.

<Table 2 here>

Shoushtari et al (2021)[59]

In this study a RT-LAMP assay for DENV2 was developed. Primers were designed using Primer-Explorer V5 software (Eiken Chemical Co. LTD, Tokyo, Japan). Incubation occurred at 65 degrees Celsius for 60 minutes. There was visual inspection as well as electrophoresis of the amplified product using ethidium bromide for fluorescence. LLOD for the assay was 100 copies per reaction. The linear range was not reported. Analytical specificity was verified by testing extracted RNA from DENV1, DENV3, DENV4, WNV, YFV, ZIKV and an extracted sample from a patient with HCV, which did not amplify. Authenticity of amplified product was not specifically assessed. Diagnostic performance of the assay was assessed using samples from 20 confirmed cases of dengue infection and 11 healthy individuals. Diagnostic sensitivity was reported to be 100% compared to a RT-qPCR assay. However, methods relating to assessment of diagnostic performance were generally unclear and diagnostic specificity data were not given. The Shoushtari assay was not used in any subsequent studies.

DISCUSSION

This review identifieds eight studies describing original serotype-specific dengue RT-LAMP assays. All assays underwent evaluation of aAll described analytical performance with some also undergoing evaluation of diagnostic process of assay design and evaluation of analytical performance, with some going on to evaluate diagnostic performance. However, sStudies were heterogenous in their design and reporting, and some omitted key experimental details. This made objective assessment and comparison of assays difficult and would likely affect attempts to replicate assays and verify findings. It is acknowledged that reports of assay development in academic literature is often a preliminary step, occurring before more rigorous efforts are made to achieve validation and accreditation. As such, authors may not be expected to fulfil requirements such as those set by CLIA for implementation of laboratory-developed tests (which were used as a template for data collection

in this study). Nevertheless, standardised assay evaluation and more detailed reporting of performance would be beneficial.

When detailed, the method of nucleic acid extraction, ingredients of the reaction mix (apart from primers) and enzymes were broadly similar across original assays. However, incubation temperature, incubation duration and method for detecting the amplified product varied considerably. LLODs and linear ranges were described for some assays and these analytical performance characteristics were comparable to those which are achievable with many RT-qPCR assays.(50) Analytical specificity was also reportedly good, with ~~very few~~virtually no incidents of non-specific amplification being reported. However, the numbers of experiments conducted using no-template controls was generally low, and subsequent studies which used the same primer sets cite non-specificity as a particular reason for modifying incubation settings, primers and/or the detection method. Some studies interpreted higher positivity by RT-LAMP (index test) when compared to RT-qPCR (reference test) as evidence of superior RT-LAMP sensitivity, when in fact this could have been due to lower RT-LAMP specificity. ~~Additionally, one study reported very high detection of mixed infection (i.e. samples positive with multiple DENV serotypes) by RT-LAMP. In other clinical studies of dengue, mixed infections detected by RT-qPCR assays were rare.[48,49]~~ Non-~~specificity is one of the risk factors reactions~~specific reactions are a feature of some nucleic acid amplification assays, including LAMP ~~are a common feature of LAMP assays in nucleic acid amplification general~~(29–31)~~., and t~~Therefore, ~~a~~ thorough assay design ~~is~~ ~~which~~ should be recognised as a priority and taken into account when considering the use of this amplification ~~technology~~chemistry ~~(for example in place of PCR, which does not suffer the same problem)~~. Further in-silico and in-vitro evaluation and modification of primer-sets may be useful, to inform and ensure their optimal performance in portable molecular diagnostic devices. Any future diagnostic accuracy studies which evaluate RT-LAMP assays (index tests) should clearly state which gold-standard assay or clinical diagnostic criteria (or composite thereof) is being used as a comparator (reference test). RT-qPCR, which is generally considered the highest performing single test for dengue infection, was the most common comparator assay in

included studies. However, if novel RT-LAMP based assays are developed which are truly portable and can be used at the ‘point-of-care’ (including sample preparation, amplification, and detection steps), then diagnostic performance of the system as a whole ~~should~~ could also be compared to ~~LFAs~~lateral flow assays, which can be used in similar settings.

Assays went on to be used in 14 subsequent studies. These included studies where samples were tested directly (i.e. without any nucleic acid extraction prior to amplification). Assays which do not need sample preparation would be of huge benefit when considering their translation into portable diagnostic devices. However, the performance of ‘direct LAMP’ and superiority of LAMP assays over PCR assays in this regard is contentious. (51) They also included studies where reagents were lyophilised or ‘printed’ into microfluidic channels and those which used novel detection methods including the use of smart phone cameras, electrochemical sensing, and sequencing (MinION). The restriction of this study to include only serotype-specific assays for detecting DENV 1-4 is a limitation, and it is acknowledged that other relevant technological advances are likely to have been made and demonstrated in other applications of LAMP-based diagnostics. Additionally, there may be other data on dengue RT-LAMP assays which have not been published or included in the Medline database~~,~~ or may have otherwise been missed by this scoping review’s search strategy.

Overall, findings from this study ~~point to~~show that serotype-specific ~~the possibility of high-performing~~ RT-LAMP assays for dengue are high-performing. ~~being used for detection and serotyping of DENV1-4.~~ When coupled with novel methods ~~of~~ for sample preparation and detection~~/authentication of the amplified product~~, these assays may ultimately lead ~~more~~ to portable molecular diagnostic devices which could be ~~beneficial~~ used across tropical and sub-tropical regions where dengue is endemic.

ACKNOWLEDGMENTS

This work was supported by the Department of Health and Social Care-funded Centre for Antimicrobial Optimisation (CAMO) at Imperial College London; and the Wellcome Trust Innovator Award (Grant ref: 215688/Z/19/Z). AH and JRM are affiliated with the NIHR Health Protection Research Unit (HPRU) in Healthcare Associated Infections and Antimicrobial Resistance at Imperial College London in partnership with the UK Health Security Agency, in collaboration with, Imperial Healthcare Partners, the University of Cambridge and the University of Warwick. The views expressed in this publication are those of the authors and not necessarily those of the NHS, the National Institute for Health Research, the Department of Health and Social Care, or the UK Health Security Agency. AH is a National Institute for Health Research (NIHR) Senior Investigator and she is Chair of the David Price Evans Global Health and Infectious Diseases Research Group at the University of Liverpool..

REFERENCES

1. Zeng Z, Zhan J, Chen L, Chen H, Cheng S. Global, regional, and national dengue burden from 1990 to 2017: A systematic analysis based on the global burden of disease study 2017. EClinicalMedicine [Internet]. 2021 Feb 1 [cited 2022 Jun 24];32. Available from: https://pubmed.ncbi.nlm.nih.gov/33681736/

2. Brady OJ, Gething PW, Bhatt S, Messina JP, Brownstein JS, Hoen AG, et al. Refining the global spatial limits of dengue virus transmission by evidence-based consensus. PLoS Negl Trop Dis [Internet]. 2012 [cited 2022 Jun 24];6(8). Available from: https://pubmed.ncbi.nlm.nih.gov/22880140/

3. Messina JP, Brady OJ, Golding N, Kraemer MUG, Wint GRW, Ray SE, et al. The current and future global distribution and population at risk of dengue. Nat Microbiol. 2019 Sep 1;4(9):1508–15.

4. Guzman MG, Gubler DJ, Izquierdo A, Martinez E, Halstead SB. Dengue infection. Nat Rev Dis Primer. 2016 Aug 18;2(1):1–25.

5. Bhatt S, Gething PW, Brady OJ, Messina JP, Farlow AW, Moyes CL, et al. The global distribution and burden of dengue. Nature. 2013 Apr 25;496(7446):504–7.

6. Montoya M, Gresh L, Mercado JC, Williams KL, Vargas MJ, Gutierrez G, et al. Symptomatic Versus Inapparent Outcome in Repeat Dengue Virus Infections Is Influenced by the Time Interval between Infections and Study Year. PLoS Negl Trop Dis [Internet]. 2013 [cited 2022 Nov 9];7(8). Available from: https://pubmed.ncbi.nlm.nih.gov/23951377/

7. De Alwis R, Williams KL, Schmid MA, Lai CY, Patel B, Smith SA, et al. Dengue Viruses Are Enhanced by Distinct Populations of Serotype Cross-Reactive Antibodies in Human Immune Sera. PLoS Pathog. 2014 Oct 1;10(10):1004386.

8. de Alwis R, Beltramello M, Messer WB, Sukupolvi-Petty S, Wahala WMPB, Kraus A, et al. In-depth analysis of the antibody response of individuals exposed to primary dengue virus infection. PLoS Negl Trop Dis [Internet]. 2011 Jun [cited 2022 Dec 12];5(6). Available from: https://pubmed.ncbi.nlm.nih.gov/21713020/

9. Lee KS, Lai YL, Lo S, Barkham T, Aw P, Ooi PL, et al. Dengue virus surveillance for early warning, Singapore. Emerg Infect Dis. 2010 May;16(5):847–9.

10. Rodriguez-Manzano J, Chia PY, Yeo TW, Holmes A, Georgiou P, Yacoub S. Improving Dengue Diagnostics and Management Through Innovative Technology. Curr Infect Dis Rep. 2018 Aug 1;20(8):1–8.

11. Muller DA, Depelsenaire ACI, Young PR. Clinical and laboratory diagnosis of dengue virus infection. J Infect Dis. 2017;215(Suppl 2):S89–95.

12. Alm E, Lindegren G, Falk KI, Lagerqvist N. One-step real-time RT-PCR assays for serotyping dengue virus in clinical samples. BMC Infect Dis [Internet]. 2015 Nov 2 [cited 2022 Nov 9];15(1). Available from: /pmc/articles/PMC4630907/

13. Morsy S, Hashan MR, Hieu TH, Mohammed AT, Elawady SS, Ghosh P, et al. The association between dengue viremia kinetics and dengue severity: A systemic review and meta-analysis. Rev Med Virol. 2020 Nov 1;30(6):1–10.

14. Manabe YC, Betz J, Jackson O, Asoala V, Bazan I, Blair PW, et al. Clinical evaluation of the BioFire Global Fever Panel for the identification of malaria, leptospirosis, chikungunya, and dengue from whole blood: a prospective, multicentre, cross-sectional diagnostic accuracy study. Lancet Infect Dis. 2022 Sep 1;22(9):1356–64.

15. Fleming KA, Horton S, Wilson ML, Atun R, DeStigter K, Flanigan J, et al. The Lancet Commission on diagnostics: transforming access to diagnostics. The Lancet. 2021 Nov 27;398(10315):1997–2050.

16. Yadav H, Shah D, Sayed S, Horton S, Schroeder LF. Availability of essential diagnostics in ten low-income and middle-income countries: results from national health facility surveys. Lancet Glob Health. 2021 Nov 1;9(11):e1553–60.

17. Notomi T, Okayama H, Masubuchi H, Yonekawa T, Watanabe K, Amino N, et al. Loop-mediated isothermal amplification of DNA. Nucleic Acids Res. 2000;28(12):e63.

18. Zhang X, Lowe SB, Gooding JJ. Brief review of monitoring methods for loop-mediated isothermal amplification (LAMP). Biosens Bioelectron. 2014 Nov 15;61:491–9.

19. Moehling TJ, Choi G, Dugan LC, Salit M, Meagher RJ. LAMP Diagnostics at the Point-of-Care: Emerging Trends and Perspectives for the Developer Community. https://doi.org/101080/1473715920211873769. 2021;21(1):43–61.

20. Moser N, Yu LS, Rodriguez Manzano J, Malpartida-Cardenas K, Au A, Arkell P, et al. Quantitative detection of dengue serotypes using a smartphone-connected handheld lab-on-chip platform. Front Bioeng Biotechnol. 2022 Sep 15;10:892853–892853.

21. Rodriguez-Manzano J, Karymov MA, Begolo S, Selck DA, Zhukov DV, Jue E, et al. Reading Out Single-Molecule Digital RNA and DNA Isothermal Amplification in Nanoliter Volumes with Unmodified Camera Phones. ACS Nano. 2016 Mar 22;10(3):3102–13.

22. Njiru ZK. Loop-mediated isothermal amplification technology: Towards point of care diagnostics. PLoS Negl Trop Dis [Internet]. 2012 Jun [cited 2022 Nov 9];6(6). Available from: https://pubmed.ncbi.nlm.nih.gov/22745836/

23. Mori A, Pomari E, Deiana M, Perandin F, Caldrer S, Formenti F, et al. Molecular techniques for the genomic viral RNA detection of West Nile, Dengue, Zika and Chikungunya arboviruses: a narrative review. Expert Rev Mol Diagn. 2021;21(6):591–612.

24. Burd EM. Validation of laboratory-developed molecular assays for infectious diseases. Clin Microbiol Rev. 2010;23(3):550–76.

25. Peeling RW, Artsob H, Pelegrino JL, Buchy P, Cardosa MJ, Devi S, et al. Evaluation of diagnostic tests: Dengue. Nat Rev Microbiol. 2010;8(12):S30–8.

26. Saah AJ, Hoover DR. “Sensitivity” and “specificity” reconsidered: The meaning of these terms in analytical and diagnostic settings. Ann Intern Med. 1997;126(1):91–4.

27. Kreitmann L, Miglietta L, Xu K, Malpartida-Cardenas K, D’Souza G, Kaforou M, et al. Next-generation molecular diagnostics: Leveraging digital technologies to enhance multiplexing in real-time PCR. TrAC Trends Anal Chem. 2023 Mar 1;160:116963.

28. Malpartida-Cardenas K, Miglietta L, Peng T, Moniri A, Holmes A, Georgiou P, et al. Single-channel digital LAMP multiplexing using amplification curve analysis. Sens Diagn. 2022;1(3):465–8.

29. Meagher RJ, Priye A, Light YK, Huang C, Wang E. Impact of primer dimers and self-amplifying hairpins on reverse transcription loop-mediated isothermal amplification detection of viral RNA. Analyst. 2018 Apr 21;143(8):1924–33.

30. Jang MJ, Kim S. Inhibition of Non-specific Amplification in Loop-Mediated Isothermal Amplification via Tetramethylammonium Chloride. Biochip J. 2022 Sep 1;16(3):326–33.

31. Özay B, McCalla SE. A review of reaction enhancement strategies for isothermal nucleic acid amplification reactions. Sens Actuators Rep. 2021 Nov 1;3:100033.

32. Tricco AC, Lillie E, Zarin W, O’Brien KK, Colquhoun H, Levac D, et al. PRISMA extension for scoping reviews (PRISMA-ScR): Checklist and explanation. Ann Intern Med. 2018;169(7):467–73.

33. Covidence - Better systematic review management [Internet]. [cited 2021 Aug 25]. Available from: https://www.covidence.org/

34. Department of Health and Human Services Centers for Medicare & Medicaid Services Centers for Disease Control and Prevention 42 CFR Part 493 Medicare, Medicaid, and CLIA Programs; Laboratory Requirements Relating to Quality Systems and Certain Personnel Qu. 2003 [cited 2022 Dec 12]; Available from: www.access.gpo/nara/index.html.

35. Bossuyt PM, Reitsma JB, Bruns DE, Gatsonis CA, Glasziou PP, Irwig L, et al. STARD 2015: An updated list of essential items for reporting diagnostic accuracy studies. The BMJ [Internet]. 2015 Oct 28 [cited 2022 Oct 21];351. Available from: /pmc/articles/PMC4623764/

36. Parida M, Horioke K, Ishida H, Dash PK, Saxena P, Jana AM, et al. Rapid detection and differentiation of dengue virus serotypes by a real-time reverse transcription-loop-mediated isothermal amplification assay. J Clin Microbiol. 2005 Jun;43(6):2895–903.

37. Neeraja M, Lakshmi V, Lavanya V, Priyanka EN, Parida MM, Dash PK, et al. Rapid detection and differentiation of dengue virus serotypes by NS1 specific reverse transcription loop-mediated isothermal amplification (RT-LAMP) assay in patients presenting to a tertiary care hospital in Hyderabad, India. J Virol Methods. 2015 Jan 1;211:22–31.

38. Hu SF, Li M, Zhong LL, Lu SM, Liu ZX, Pu JY, et al. Development of reverse-transcription loop-mediated isothermal amplification assay for rapid detection and differentiation of dengue virus serotypes 1-4. BMC Microbiol [Internet]. 2015 [cited 2022 Dec 12];15(1). Available from: https://pubmed.ncbi.nlm.nih.gov/26572227/

39. Lau YL, Lai MY, Teoh BT, Abd-Jamil J, Johari J, Sam SS, et al. Colorimetric detection of dengue by single tube reverse-transcription-loop-mediated isothermal amplification. PLoS ONE [Internet]. 2015 Sep 18 [cited 2022 Feb 11];10(9). Available from: https://pubmed.ncbi.nlm.nih.gov/26384248/

40. Yaren O, Alto BW, Gangodkar PV, Ranade SR, Patil KN, Bradley KM, et al. Point of sampling detection of Zika virus within a multiplexed kit capable of detecting dengue and chikungunya. BMC Infect Dis [Internet]. 2017 Apr 20 [cited 2022 Dec 12];17(1). Available from: https://pubmed.ncbi.nlm.nih.gov/28427352/

41. Kim JG, Baek SH, Kim S, Kim HI, Lee SW, Phan LMT, et al. Rapid discriminative detection of dengue viruses via loop mediated isothermal amplification. Talanta. 2018 Dec 1;190:391–6.

42. Lopez-Jimena B, Bekaert M, Bakheit M, Frischmann S, Patel P, Simon-Loriere E, et al. Development and validation of four one-step real-time RT-LAMP assays for specific detection of each dengue virus serotype. PLoS Negl Trop Dis. 2018;12(5):1–22.

43. Shoushtari M, Salehi-Vaziri M, Roohvand F, Arashkia A, Jalali T, Azadmanesh K. Taguchi array optimization of the reverse transcription loop-mediated isothermal amplification (RT-LAMP) assay for sensitive and rapid detection of dengue virus serotype 2. Biotechnol Lett. 2021 Nov 1;43(11):2149–60.

44. Yamagishi J, Runtuwene LR, Hayashida K, Mongan AE, Thi LAN, Thuy LN, et al. Serotyping dengue virus with isothermal amplification and a portable sequencer. Sci Rep [Internet]. 2017 Dec 1 [cited 2022 Dec 12];7(1). Available from: https://pubmed.ncbi.nlm.nih.gov/28615658/

45. Ganguli A, Ornob A, Yu H, Damhorst GL, Chen W, Sun F, et al. Hands-free smartphone-based diagnostics for simultaneous detection of Zika, Chikungunya, and Dengue at point-of-care. Biomed Microdevices [Internet]. 2017 Dec 1 [cited 2022 Dec 12];19(4). Available from: https://pubmed.ncbi.nlm.nih.gov/28831630/

46. Minero GAS, Nogueira C, Rizzi G, Tian B, Fock J, Donolato M, et al. Sequence-specific validation of LAMP amplicons in real-time optomagnetic detection of Dengue serotype 2 synthetic DNA. Analyst. 2017 Sep 21;142(18):3441–50.

47. Priye A, Bird SW, Light YK, Ball CS, Negrete OA, Meagher RJ. A smartphone-based diagnostic platform for rapid detection of Zika, chikungunya, and dengue viruses. Sci Rep [Internet]. 2017 Mar 20 [cited 2022 Feb 11];7. Available from: /pmc/articles/PMC5357913/

48. Hin S, Lopez-Jimena B, Bakheit M, Klein V, Stack S, Fall C, et al. Fully automated point-of-care differential diagnosis of acute febrile illness. PLoS Negl Trop Dis. 2021 Feb 1;15(2):1–24.

49. Kumar S, Sharma S, Kumari S, Pande V, Savargaonkar D, Anvikar AR. Magnetic Multiplex Loop Mediated Isothermal Amplification (MM-LAMP) technique for simultaneous detection of dengue and chikungunya virus. J Virol Methods [Internet]. 2022 Feb 1 [cited 2022 Dec 12];300. Available from: https://pubmed.ncbi.nlm.nih.gov/34896457/

50. Songjaeng A, Thiemmeca S, Mairiang D, Punyadee N, Kongmanas K, Hansuealueang P, et al. Development of a Singleplex Real-Time Reverse Transcriptase PCR Assay for Pan-Dengue Virus Detection and Quantification. Viruses [Internet]. 2022 Jun 1 [cited 2022 Dec 12];14(6). Available from: https://pubmed.ncbi.nlm.nih.gov/35746742/

51. Wilson-Davies ESW, Mahanama AIK, Samaraweera B, Ahmed N, Friar S, Pelosi E. Concerns regarding the sensitivity of the OptiGene direct SARS-CoV-2 LAMP assay and its suitability for use in at-risk groups and hospital staff. J Infect. 2021 Feb 1;82(2):282–327.

SUPPORTING INFORMATION CAPTIONS

S1 Table: PRISMA-ScR checklist

S1 Data: – Data collected from individual papers describing original RT-LAMP assays

FUNDING

~~This work was funded by the Wellcome Trust (grant number 215688/Z/19/Z)~~

PA is funded by the Wellcome Trust (ref: 215688/Z/19/Z). KMP is funded by NIHR (ref: NIHR134694). KHC is funded by the Wellcome Trust (ref: 226691/Z/22/Z). PG and JRM are funded by Imperial College London. AH is funded by Imperial College London and the University of Liverpool. DM, AS and PAvirutan are funded by Mahidol University. The funders had no role in study design, data collection and analysis, decision to publish, or preparation of the manuscript.

COMPETING INTERESTS

All authors declare there are no competing interests

Table 1: Search terms

1	exp Dengue/ or exp Dengue Virus/
2	dengue.mp.
3	loop mediated.mp.
4	isothermal amplification.mp.
5	LAMP.mp.
6	1 or 2
7	3 or 4 or 5
8	6 and 7

Table 2: Summary of studies describing original RT-LAMP assays for dengue virus serotypes 1-4

**Publication (year)**	**Assay details**					**Analytical performance**			**Diagnostic performance**				**Subsequent publications (year)**
	**Extraction method**	**Reaction mixture**	**Incubation**	**Detection**	**Target (gene)**	**Lower limit-of-detection**	**Specificity: other organisms**	**Specificity: examination of amplified product**	**Specimens**	**Reference standard**	**Sensitivity**	**Specificity**	
Parida et al (2005)	QIAamp viral RNA mini kit (QIAGEN, Hilden Germany)	Loopamp DNA amplification kit (Eiken Chemical Co. Ltd., Japan)	63.0 degrees Celsius for 60 mins (but determined positive at 30 mins)	Visual inspection (+/- addition of SYBR Green I) and real-time monitoring of turbidity and agarose gel analysis*	DENV1 (3' ~~NCR~~UTR)<br>DENV2 (3' UT~~NC~~R)<br>DENV3 (3' UT~~NC~~R)<br>DENV4 (3' UT~~NC~~R)	1 PFU/ml (=2.5x10^-2 PFU/reaction)<br>0.1 PFU/ml (=2.5x10^-3 PFU/reaction)<br>0.1 PFU/ml (=2.5x10^-3 PFU/reaction)<br>0.1 PFU/ml (=2.5x10^-3 PFU/reaction)	JEV, WNV or SLEV templates - no amplification	Restriction enzyme digestion + agarose gel electrophoresis - product sizes in good agreement with predicted. Further confirmation with sequencing - nucleotide sequences matched target	83 serum samples:<br>- 25 confirmed dengue cases<br>- 38 suspected dengue cases<br>- 20 healthy individuals	Considered positive if: Conventional RT-PCR (+) OR nested RT-PCR (+) OR virus isolation (+)	31/31 (100.0%)	20/20 (100.0%)	Li et al (2011)<br>Chagan-Yasutan et al (2013)<br>Lo et al (2013)<br>Yamagishi et al (2017)<br>Kumar et al (2022)<br>Gaber et al (2022)
Neeraja et al (2015)	QIAamp viral RNA mini kit (QIAGEN, Hilden Germany)	Isothermal Master Mix ISO-001 (Optigene, U.K.)	63.0 degrees Celsius for 35 minutes	Visual inspection (+/- addition of SYBR Green I) and agarose gel analysis*	DENV1 (NS1)<br>DENV2 (NS1)<br>DENV3 (NS1)<br>DENV4 (NS1)	N/A	Other flaviviruses (sic) including JEV, WNV, HCV and CHIKV - no amplification	Restriction enzyme digestion + agarose gel electrophoresis - product sizes in good agreement with predicted. Further confirmation using sequencing - results not reported in manuscript	300 serum or plasma samples:<br>- 250 dengue cases<br>- 50 healthy individuals	Considered positive if: RT-qPCR (+)	140/140 (100.0%)**	152/160 (95.0%)**	Dave et al (2022)
Hu et al (2015)	QIAamp viral RNA mini kit (QIAGEN, Hilden Germany)	Bespoke mix	63.0 degrees Celsius for 45 minutes	Visual inspection (+/- addition of SYBR Green I) and real-time monitoring of flourescence (SYBR Green I) and agarose gel analysis*	DENV1 (NS2A)<br>DENV2 (NS4B)<br>DENV3 (NS4A)<br>DENV4 (3'UTR)	1x10^1 copies/uL (=2.5x10^2 copies/reaction)<br>1x10^1 copies/uL (=2.5x10^2 copies/reaction)<br>1x10^1 copies/uL (=2.5x10^2 copies/reaction)<br>1x10^1 copies/uL (=2.5x10^2 copies/reaction)	JEV, YFV, HSV and Epstein-Barr virus x10 times - no amplification	Restriction enzyme digestion + agarose gel electrophoresis - images shown in manuscript. Further confirmation using sequencing - 'specificity of amplification confirmed'	210 serum samples:<br>- 190 confirmed dengue cases<br>- 20 healthy individuals	Considered positive if: 'confirmed to be infected by dengue by clinical diagnosis'	50/50 (100.0%)<br>59/60 (98.3%)<br>40/40 (100%)<br>39/40 (97.5%)	20/20 (100.0%)<br>20/20 (100.0%)<br>20/20 (100%)<br>20/20 (100%)	Ganguli et al ()

Lau et al (2015)	QIAamp viral RNA mini kit (QIAGEN, Hilden Germany)	Loopamp RNA amplification kit (Eiken Chemical Co. Ltd., Japan)	65.0 degrees Celsius for 30 min (DENV1-3 assays) or 45 min (DENV4 assay)	Visual inspection (+ HNB dye, Sigma, USA) and real-time monitoring of turbidity*	DENV1 (3' NCR)<br>DENV2 (3' NCR)<br>DENV3 (3' NCR)<br>DENV4 (3' NCR)	The detection limit of RT-LAMP for 3'-NCR was as low as ten copies (=2.5x10^2 copies/reaction)	JEV, CHIKV and Sindbis virus - no amplification	Agarose gel electrophoresis - typical DNA ladder observed (though analysis of amplicon size using restriction enzyme not reported)	213 serum samples<br>- 189 suspected dengue cases<br>- 24 healthy individuals	Considered positive if 2 or more of the following were true: RT-qPCR (+), ELISA (+), RT-LAMP (+).	115/115 (100%)	98/98 (100%)	Minero et al (2017)<br>Priye et al (2017)<br>Meagher et al (2018)<br>Sigera et al (2019)
Yaren et al (2017)	Unclear	Bespoke mix	65.0 degrees Celsius for 60-90 minutes	Real-time detection of fluorescence (TAMARA-labelled LB or LF probe) then modification to include 'target specific strand-displaceable probe' (fluorescence detected by cell phone camera).	DENV1 ~~()~~(NS5)	1.22 PFU per assay (=1.22x10^0 PFU/reaction)	ZIKV and CHIKV RNA - no amplification	Agarose gel electrophoresis - typical DNA ladder observed (though analysis of amplicon size using restriction enzyme not reported)	N/A	N/A	N/A	N/A	Yaren et al (2018)
Kim et al (2018)	QIAamp viral RNA mini kit (QIAGEN, Hilden Germany)	Bespoke mix	69.7 (DENV1 assay), 65.0 (DENV2 assay) or 66.5 (DENV4 assay) degrees Celsius for 40 minutes	Visual inspection (UV light illumination)	DENV1 (E)<br>DENV2 (NS1)<br>DENV4 (PrM)	33 copies / uL (=8.25x10^2 copies/reaction)<br>3.55 copies / uL (=8.88x10^1 copies/reaction)<br>9.06 copies / uL (2.27x10^2 copies/reaction)	DENV3, norovirus, rotavirus and bovine viral diarrhea - no amplification	Agarose gel electrophoresis - typical DNA ladder observed (though analysis of amplicon size using restriction enzyme not reported)	N/A	N/A	N/A	N/A	
Lopez-Jimena et al (2018)	Various commercially available extraction methods depending on source of samples/viruses	Bespoke mix	64.0 degrees Celsius for 45 min (DENV1 assay), 90 min (DENV2 assay), 75 min (DENV3 assay) or 50 min (DENV4 assay)	Real-time detection of fluorescence	DENV1 (various)<br>DENV2 (various)<br>DENV3 (various)	22 RNA molecules per reaction (=2.2x10^1 copies/reaction)<br>542 RNA molecules per reaction (=5.42x10^2 copies/reaction)<br>92 RNA molecules per reaction (9.2x10^1 copies/reaction)	ZIKV, YFV, WNV, Ntaya virus, *S. typhi*, *S. paratyphi*, *S. pneumoniae* and *P. falciparum* - no amplification	Melting curve analysis - single peak temperatures indicated specific amplification	78 samples:<br>- 42 imported blood/serum samples<br>- 36 imported RNA extracts	Considered positive if: RT-qPCR (+)	Initially sensitivity = 17/24 (70.8%), then false-negative samples re-extracted re-run and sensitivity = 23/24 (95.8%)**	7/7 (100%)**	Hin et al (2021)

					DENV4 (various)	197 RNA molecules per reaction (=1.97x10^2 copies/reaction)							
Shoushtari et al (2021)	QIAamp viral RNA mini kit (QIAGEN, Hilden Germany)	Bespoke mix	65.0 degrees Celsius for 60 minutes	Agarose gel analysis	DENV2 (C-PrM)	100 RNA copies per reaction (=1x10^2 copies/reaction)	DENV1, DENV3, DENV4, WNV, YFV, ZIKV RNA (and serum from hepatitis C patient) - no amplification	N/A	31 serum samples<br>- 20 dengue cases<br>- 11 healthy individuals	Considered positive if: RT-qPCR (+)	15/15 (100%)	Results for 11 healthy sera not presented	

Abbreviations: DENV = dengue virus, PFU = plaque forming units, JEV = Japanese encephalitis virus, WNV = West Nile virus, SLE = St Louis encephalitis virus, HCV = hepatitis C virus, CHIKV = chikungunya virus, HSV = herpes simplex virus, ZIKV = Zika virus, RT-PCR = reverse-transcriptase polymerase chain reaction, ELISA = enzyme-linked immunosorbent assay, RT-LAMP = reverse transcriptase loop-mediated isothermal amplification

* Studies frequently described more than one method for detecting amplified products of RT-LAMP. However, it was sometimes not clear how discrepant results were handled in analysis of assay analytical and diagnostic performance
** Multiple alternative analyses are reported in the manuscript

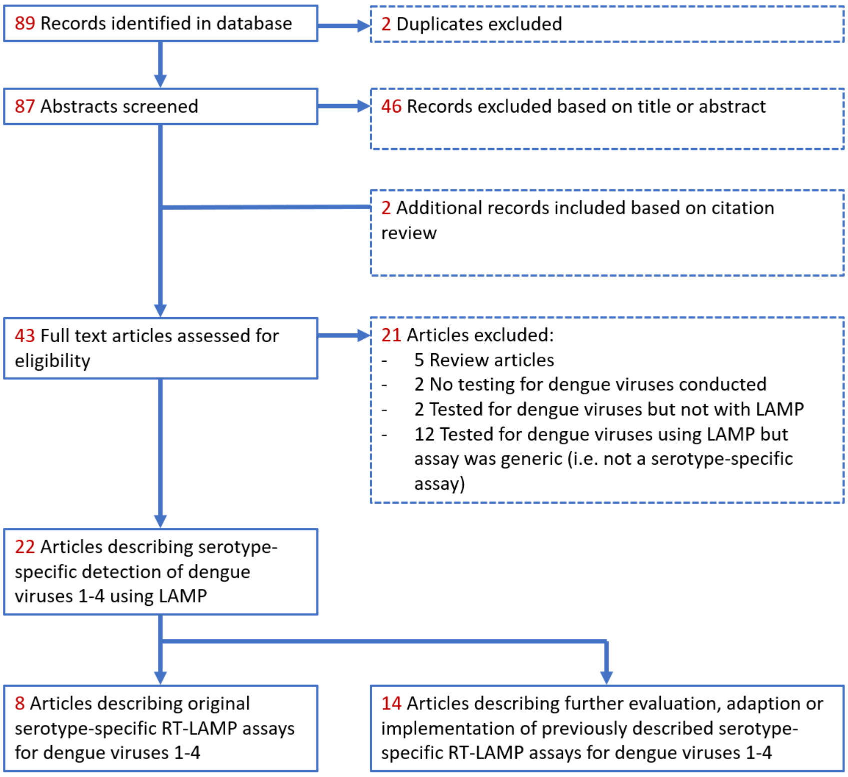

Figure 1: ~~Consort diagram~~Workflow showing the assessment of articles and their inclusion in this review

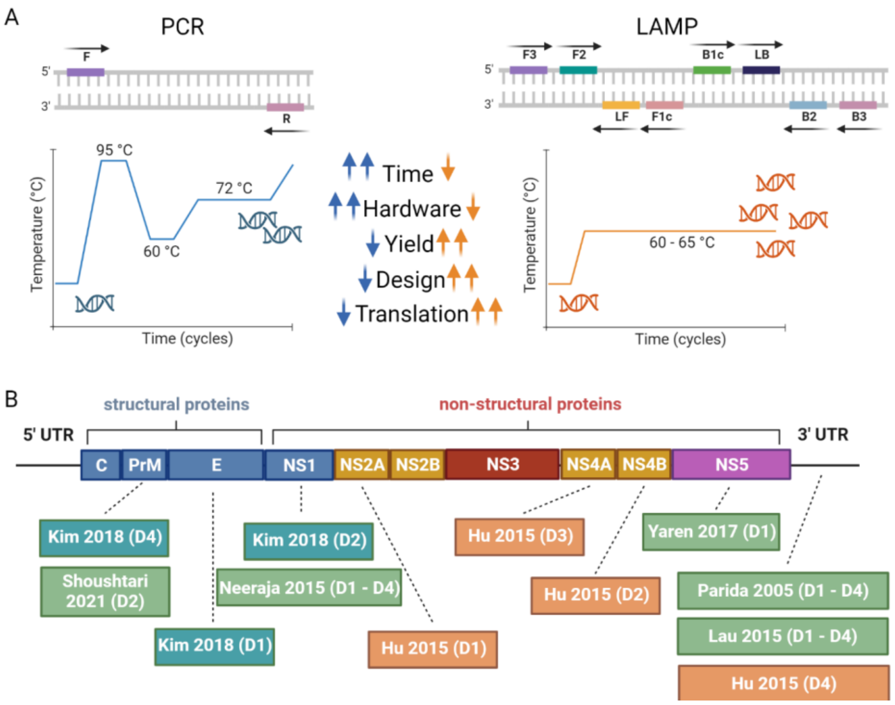

Figure 2: (A) Comparison between polymerase chain reaction (PCR) and loop-mediated isothermal amplification (LAMP). Primer binding regions are shown at the top, thermal cycling conditions at the bottom, and differences between the methods in the text where arrows in orange refers to LAMP and in blue to PCR. (B) Schematic of dengue genome showing the position of primer-binding in published serotype-specific RT-LAMP assays.

Dr Paul Arkell
Centre for Antimicrobial Optimisation
Department of Infectious Diseases
Imperial College London

Gathsaurie Neelika Malavige
Academic Editor
PLOS Global Public Health

Dear PLOS Global Public Health Editorial Team,

Re: Analytical and diagnostic performance characteristics of reverse-transcriptase loop-mediated isothermal amplification assays for dengue serotypes 1-4: a scoping review to inform potential use in portable molecular diagnostic devices

Thank you for your email dated 10th May 2023. Please find our responses to reviewers' comments below and a revised manuscript attached.

Please do not hesitate to contact me if anything else is required.

Yours faithfully,

Dr Paul Arkell

Journal Requirements:

1. Please amend your detailed Financial Disclosure statement. This is published with the article. It must therefore be completed in full sentences and contain the exact wording you wish to be published.

a. State the initials, alongside each funding source, of each author to receive each grant.

b. State what role the funders took in the study. If the funders had no role in your study, please state: "The funders had no role in study design, data collection and analysis, decision to publish, or preparation of the manuscript."

c. If any authors received a salary from any of your funders, please state which authors and which funders.

This information has been included

2. Please provide separate figure files in .tif or .eps format.

This is now included

3. Please review your reference list to ensure that it is complete and correct. If you have cited papers that have been retracted, please include the rationale for doing so in the manuscript text, or remove these references and replace them with relevant current references. Any changes to the reference list should be mentioned in the rebuttal letter that accompanies your revised manuscript. If you need to cite a retracted article, indicate the article's retracted status in the References list and also include a citation and full reference for the retraction notice.

Reference list has been reviewed

Reviewers' comments:

Reviewer #1: Greetings!! Method in Abstract: Study design and duration of the study are absent. The authors also should mention the duration of the study in method in the main article. There are some grammatical mistake in some lines otherwise the article is nicely written.

Thank you, we have added the study design and dates to the abstract and the methods

Reviewer #2: 1. This is a very useful paper. But the text is excessive. The manuscript needs to be condensed some more. The word count now is nearly 6000 and this is too much considering the subject matter.

Thank you for this suggestion. We have considerably reduced the wordcount. This has mostly been achieved through summarising each 'study describing original RT-LAMP assay', and removing the 'narrative' section.

2. It would be helpful for the reader to have a diagram that explains the third section - LAMP as a potential solution - and shows how it is different from RT-PCR.

Thank you. We have added a figure which outlines how LAMP primers bind to target DNA and how the reaction proceeds.
