# Supplementary material for: Analytical and diagnostic performance characteristics of reverse-transcriptase loop-mediated isothermal amplification assays for dengue virus serotypes 1–4: A scoping review to inform potential use in portable molecular diagnostic devices
Source: PLOS Glob Public Health. 2023 Aug 8;3(8):e0002169. doi: 10.1371/journal.pgph.0002169 (PMC10409275; doi:10.1371/journal.pgph.0002169)
Supplement: Attachment — Submitted filename: Response to PGPH reviewers.docx [file pgph.0002169.s003.docx]

Dr Paul Arkell
Centre for Antimicrobial Optimisation
Department of Infectious Diseases
Imperial College London

Gathsaurie Neelika Malavige
Academic Editor
PLOS Global Public Health

Dear PLOS Global Public Health Editorial Team,

**Re: Analytical and diagnostic performance characteristics of reverse-transcriptase loop-mediated isothermal amplification assays for dengue serotypes 1-4: a scoping review to inform potential use in portable molecular diagnostic devices**

Thank you for your email dated 10^th^ May 2023. Please find our responses to reviewers’ comments below and a revised manuscript attached.

Please do not hesitate to contact me if anything else is required.

Yours faithfully,

Dr Paul Arkell

**Journal Requirements:**

1. Please amend your detailed Financial Disclosure statement. This is published with the article. It must therefore be completed in full sentences and contain the exact wording you wish to be published.

a. State the initials, alongside each funding source, of each author to receive each grant.

b. State what role the funders took in the study. If the funders had no role in your study, please state: “The funders had no role in study design, data collection and analysis, decision to publish, or preparation of the manuscript.”

c. If any authors received a salary from any of your funders, please state which authors and which funders.

This information has been included

2. Please provide separate figure files in .tif or .eps format.

This is now included

3. Please review your reference list to ensure that it is complete and correct. If you have cited papers that have been retracted, please include the rationale for doing so in the manuscript text, or remove these references and replace them with relevant current references. Any changes to the reference list should be mentioned in the rebuttal letter that accompanies your revised manuscript. If you need to cite a retracted article, indicate the article’s retracted status in the References list and also include a citation and full reference for the retraction notice.

Reference list has been reviewed

**Reviewers' comments:**

Reviewer #1: Greetings!! Method in Abstract: Study design and duration of the study are absent. The authors also should mention the duration of the study in method in the main article. There are some grammatical mistake in some lines otherwise the article is nicely written.

Thank you, we have added the study design and dates to the abstract and the methods

Reviewer #2: 1. This is a very useful paper. But the text is excessive. The manuscript needs to be condensed some more. The word count now is nearly 6000 and this is too much considering the subject matter.

Thank you for this suggestion. We have considerably reduced the wordcount. This has mostly been achieved through summarising each ‘study describing original RT-LAMP assay’, and removing the ‘narrative’ section.

2. It would be helpful for the reader to have a diagram that explains the third section - LAMP as a potential solution - and shows how it is different from RT-PCR.

Thank you. We have added a figure which outlines how LAMP primers bind to target DNA and how the reaction proceeds.
